# Nanopore DNA Sequencing and Genome Assembly on the International Space Station

**DOI:** 10.1038/s41598-017-18364-0

**Published:** 2017-12-21

**Authors:** Sarah L. Castro-Wallace, Charles Y. Chiu, Kristen K. John, Sarah E. Stahl, Kathleen H. Rubins, Alexa B. R. McIntyre, Jason P. Dworkin, Mark L. Lupisella, David J. Smith, Douglas J. Botkin, Timothy A. Stephenson, Sissel Juul, Daniel J. Turner, Fernando Izquierdo, Scot Federman, Doug Stryke, Sneha Somasekar, Noah Alexander, Guixia Yu, Christopher E. Mason, Aaron S. Burton

**Affiliations:** 1 Biomedical Research and Environmental Sciences Division, NASA Johnson Space Center, Houston, TX United States; 2 Department of Laboratory Medicine, University of California San Francisco, San Francisco, CA United States; 3 UCSF-Abbott Viral Diagnostics and Discovery Center, San Francisco, CA United States; 4 NASA Postdoctoral Program, NASA Johnson Space Center, Houston, TX United States; 5 JES Tech, Houston, TX United States; 6 Astronaut Office, NASA Johnson Space Center, Houston, TX United States; 7 Department of Physiology and Biophysics, Weill Cornell Medicine, New York, NY USA; 8 Solar System Exploration Division, NASA Goddard Space Flight Center, Greenbelt, MD United States; 9 Exploration Systems Projects Office, NASA Goddard Space Flight Center, Greenbelt, MD United States; 10 Space Biosciences Division, NASA Ames Research Center, Moffett Field, CA United States; 11 Formerly JES Tech, Houston, TX United States; 12 Applied Engineering and Technology Directorate, NASA Goddard Space Flight Center, Greenbelt, MD 20771 United States; 13 Oxford Nanopore Technologies, Oxford, UK; 14 The HRH Prince Alwaleed Bin Talal Bin Abdulaziz Alsaud Institute for Computational Biomedicine, Weill Cornell Medicine, New York, NY USA; 15 The Feil Family Brain and Mind Research Institute, Weill Cornell Medicine, New York, NY USA; 16 Astromaterials Research and Exploration Science Division, NASA Johnson Space Center, Houston, TX United States

## Abstract

We evaluated the performance of the MinION DNA sequencer in-flight on the International Space Station (ISS), and benchmarked its performance off-Earth against the MinION, Illumina MiSeq, and PacBio RS II sequencing platforms in terrestrial laboratories. Samples contained equimolar mixtures of genomic DNA from lambda bacteriophage, *Escherichia coli* (strain K12, MG1655) and *Mus musculus* (female BALB/c mouse). Nine sequencing runs were performed aboard the ISS over a 6-month period, yielding a total of 276,882 reads with no apparent decrease in performance over time. From sequence data collected aboard the ISS, we constructed directed assemblies of the ~4.6 Mb *E. coli* genome, ~48.5 kb lambda genome, and a representative *M. musculus* sequence (the ~16.3 kb mitochondrial genome), at 100%, 100%, and 96.7% consensus pairwise identity, respectively; *de novo* assembly of the *E. coli* genome from raw reads yielded a single contig comprising 99.9% of the genome at 98.6% consensus pairwise identity. Simulated real-time analyses of in-flight sequence data using an automated bioinformatic pipeline and laptop-based genomic assembly demonstrated the feasibility of sequencing analysis and microbial identification aboard the ISS. These findings illustrate the potential for sequencing applications including disease diagnosis, environmental monitoring, and elucidating the molecular basis for how organisms respond to spaceflight.

## Introduction

Durations for Mars missions are likely to range from 1.5 to 3 years, with 12 to 24 months of that time spent in transit between the planets, based on current propulsion technologies and planetary orbital dynamics. In response to spaceflight, the human immune response becomes dysregulated^1^, and microbial pathogenicity can increase during spaceflight^2^. Beyond gene expression-mediated virulence changes, it is unclear how microbial populations would evolve, both in terms of population ecology and genetic mutations, over the course of a multi-year mission with increased exposure to ionizing radiation and microgravity during transit. This ongoing microbial evolution could have a profound impact on crew health, as microbiome stability and dynamics are known to have significant effects on human health on Earth^3,4^. Considering the time required to reach Mars, intervention from Earth during the course of a Mars mission will be limited to electronic communication, meaning that any analyses or monitoring to be performed must be done *in situ*. There is also a clear need for in-flight clinical diagnostic capability to ensure that any infections can be managed appropriately, including the administration of targeted antimicrobials.

Sequencing is a technology that could potentially address several critical spaceflight needs: infectious disease diagnosis, population metagenomics, gene expression changes, and accumulation of genetic mutations. Based on size, power, and ease of use considerations, the MinION™ DNA sequencer (Oxford Nanopore Technologies, Oxford, UK) was the most spaceflight-ready of commercially available sequencers. This device sequences DNA and RNA by measuring current changes caused by nucleic acid molecules passing through protein nanopores embedded in membranes; the change in current is diagnostic of the sequence of the DNA or RNA occupying the pore at a given time. In order to evaluate the performance of the MinION in a space environment, we tested it aboard the International Space Station (ISS). Orbiting 400 km above the Earth and travelling at 28,000 km/h, the ISS is in constant freefall and maintains a continuous microgravity environment. Although the MinION has been successfully reported to operate in remote locations on Earth^5–8^, operation aboard the ISS poses additional challenges, including possible flow cell membrane disruption due to launch shocks and vibrations, difficulty in removing air bubbles that form during sample handling, along with a potential increase in susceptibility to those air bubbles introduced to the flow cell during loading in microgravity; air bubbles in particular can damage the flow cell membranes or nanopores, and can also block the nanopores directly. In parabolic flight testing of the nanopore sequencer, we obtained only three reads while airborne^9^. From this experience, we identified a number of procedural changes in order to improve performance in flight and enable the present work (see Materials and Methods).

Here we describe the results of nanopore DNA sequencing experiments performed aboard the ISS with samples containing an equimolar mixture of genomic DNA extracted from a virus (*Enterobacteria phage lambda*), a bacterium (*Escherichia coli*), and a model mammalian organism, the mouse (*Mus musculus*). In parallel, we performed control experiments on the ground and made cross-platform comparisons with genomic sequence data obtained from the same samples on Illumina MiSeq (Illumina, San Diego, CA) and PacBio (Pacific Biosciences, Menlo Park, CA) instruments. Simulated analyses of in-flight data using an automated metagenomic pipeline and *de novo* assembly algorithms demonstrate the feasibility of on-board, real-time analysis and genomic assembly for future sequencing applications in space.

## Results

### Flight and Ground Control sequencing with the MinION

**Figure 1:**
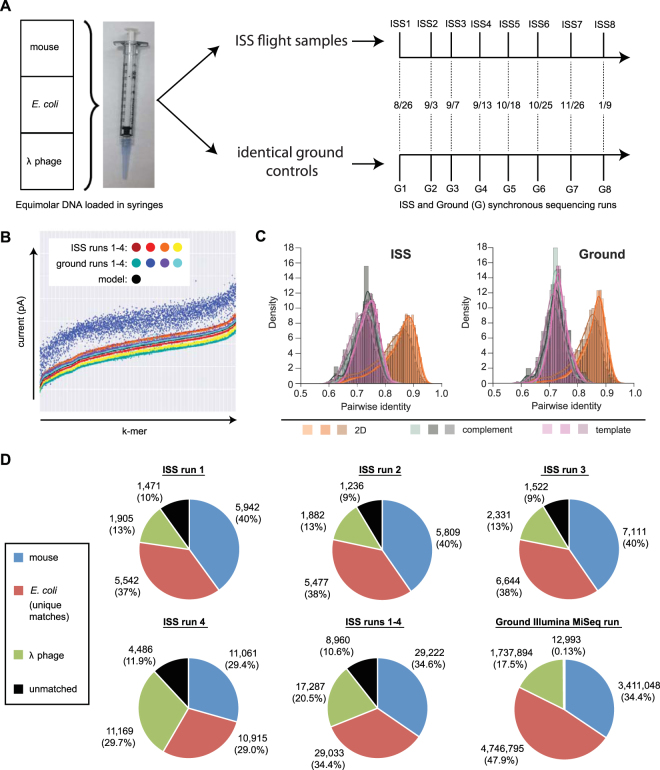
Study design and flight/ground nanopore performance. (**A**) A mixture of equimolar DNA from mouse, *E. coli* and lambda phage genomes was sequenced in parallel on Earth (“Ground”) and in-flight on the ISS (after being delivered by a SpaceX Dragon capsule; flow cells were shipped to the Kennedy Space Center on July 11, 2016). Synchronous nanopore sequencing runs were performed from August 26, 2016 to January 9, 2017. (**B**) Plot of mean current intensity in picoAmperes (pA; Y-axis) against k-mers (x-axis) in order of increasing mean current based on a model distribution from Oxford Nanopore Technologies (black). Current distributions are tightly clustered with the exception of lower-quality ground #2. (**C**) Comparison of pairwise identities of aligned reads between ISS runs 1–4 and ground runs 1–4. (**D**) Pie charts of the read distributions corresponding to each ISS run and pooled ISS runs 1–4.

Nine sequencing experiments were conducted aboard the ISS between August 26, 2016 and January 9, 2017 (Fig. 1A, Supplementary Table 1). Identical, simultaneous sequencing runs were also performed on the ground. For eight of the nine sequencing runs, a frozen sample containing ready-to-sequence DNA libraries was thawed and loaded on a new MinION flow cell, and the run was initiated using MinKNOW software (Oxford Nanopore Technologies, Inc. v0.51); for run 6, an initial 6 hour sequencing run was performed, after which a second thawed library was loaded on the flow cell and a 48 hour sequencing run was initiated (ISS6.2). All samples contained equimolar inputs of lambda, *E. coli* and mouse genomic DNA. Of the eighteen combined flight and ground experiments, all but two ground experiments (G2 and G6.2) produced good yields of high-quality sequencing data. The cause for the poor performance in G2 is not definitely known, although the low number of available pores suggested disruption of the nanopores during handling, shipping, or storage. For G6.2, the reduced performance was not unexpected because the flow cell appeared to have relatively fewer active pores prior to its first use (G6.1), and then was re-used without rinsing with the manufacturer’s recommended wash buffer; similarly, it was observed that ISS6.2 also had a decrease in available pores from ISS6.1. Two of the runs, G7 and ISS7, terminated earlier than expected. In the case of G7, the wireless adapter on the Surface Pro 3 was left on and the combined power consumption of the MinION and wireless card exceeded the charging capacity of the Surface Pro 3, causing it to power off; for ISS7, the power cord became disconnected at some point during the sequencing run and the Surface Pro 3 shut off when its battery ran out of power. Because sequence data files are written as each molecule is sequenced, the only consequence of these early terminations was a reduction in the total number of molecules sequenced. Upon completion of sequencing experiments in-flight, all FAST5/HDF files produced were downloaded from the ISS to Earth. Data transfers for each sequencing run (4–20 Gb of data distributed among 15,000 to 61,000 files) took between 1 and 6 hours. The flight and ground data were then analyzed using a number of open-source and custom-developed bioinformatic workflows (see Materials and Methods).

A key determinant of the success or failure of sequencing on the MinION is the number of active pores identified during the MUX scan performed at the initiation of sequencing. An active pore is one where current can be measured going through the pore. Each flow cell contains a total of 2,048 nanopores, each of which is capable of sequencing molecules that pass through it. In practice, however, a subset of these nanopores will fail at some point during manufacture, transit, handling, storage or use, reducing the total number of active pores. Vibration testing on the ground suggested that ~70% of the nanopores in the R7 flow cells, as determined by platform QC analysis, should be active after launch vibration^9^. Although there was considerable variability in the number of active pores between the 16 flow cells used in this study, no statistically significant decrease in the number of active pores in the flow cells used on the ISS was observed compared with the ones used on the ground (Supplementary Table 1). Across all 9 runs, a total of ~284,000 reads were generated, as compared to ~130,000 from the ground controls. Thus, to a first approximation, MinION sequencing performance on the ISS was comparable to or better than MinION sequencing on the ground.

Samples were prepared for sequencing using the 2D sequencing library preparation kit (Oxford Nanopore Technologies). This process adds a hairpin adapter to duplex DNA, allowing both strands to be sequenced (2D reads) to improve sequence accuracy; reads containing data from only one of the strands are also generated (1D reads). Initial quality control of the data was conducted using a custom Metrichor workflow for 1D and 2D basecalling and species identification (Supplementary Figure 1). We performed additional characterization of the in-flight and ground reads by analyzing the *k*-mer (the number of nucleotides that occupy the nanopore at a given time, causing observable current changes; in this case *k* = 6) current plots across the first four sets of runs (Fig. 1B), as well as measurements of signal parsing quality including gaps in the detection of signal conductance (skips) and signal changes that do not manifest in a basecalled sequence (stays) per base (Supplementary Figure 2). With the exception of G2, the *k*-mer current profiles, skips/base, and stays/base were within the range of expected values for MinION sequencing and permitted adequate basecalling. We next analyzed the 1D and 2D accuracies of the first four in-flight and ground reads, along with organism identification from the read data (Fig. 1C, 1D, Supplementary Figures 1 and 3). In general, read accuracies across the ISS and ground runs were comparable, spanning 74–80% (ISS) and 71–78% (ground) median base accuracy for the 1D reads and 83–92% (ISS) and 80–90% (ground) median base accuracy for the 2D reads, although in most cases the accuracies of the in-flight reads were slightly higher. The distributions of reads in both the flight and ground data were found to be fairly evenly distributed across the *E. coli* genome (Supplementary Figure 4), with median read lengths were typically 5,000–6,000 bases (Supplementary Figure 5). The elevated coverage of the *E. coli* genome near 0.5 Mb (Supplementary Figure 4) is likely an artifact from the integration of the rated lambda genome into the *E. coli* genome; we are thus unable to distinguish *E. coli* reads from integrated lambda genome versus bacteriophage lambda reads.

### Benchmarking of the MinION sequencing data against MiSeq and PacBio RSII data

To assess the accuracy of the nanopore MinION data and to establish “gold standard” genomic references for the lambda and *E. coli* genomes, we used the same stock of DNA for replicate sequencing experiments on PacBio RSII and Illumina MiSeq instruments. We ran 22 SMRT cells across independently sequenced lambda, *E. coli*, and mouse genomic DNA samples on the RSII (1.1 M reads with 11,366 base pair (bp) average length) and a full flow cell on the MiSeq (21,214,638 single-end 160 bp reads). The MiSeq run consisted of separately indexed samples corresponding to genomic DNA from lambda (792,838 reads), *E. coli* (3,189,286 reads), mouse (7,197,511 reads), as well as an equimolar mixture of genomic DNA from all 3 organisms (10,035,003 reads) that replicated the nanopore-sequenced samples. *De novo* assembly of the *E. coli* genome using the Hierarchical Genome Assembly Process (HGAP, v2) from PacBio reads at 162.7X coverage generated a single contiguous sequence (contig) of size 4,734,145 bp (Supplementary Figure 6). *De novo* assembly from all *E. coli* reads on the Illumina MiSeq run, identified by alignment to the most closely matched genome in the National Center for Biotechnology (NCBI) nucleotide (nt) database (*E. coli* K-12, CP014348), produced 245 contigs with coverage of 80.1% of the genome at 99.7% identity to the PacBio reference genome (Supplementary Figure 7), while directed assembly of the *E. coli* Illumina reads to the PacBio reference genome resulted in 99.0% coverage at 99.97% identity. Directed assembly of the viral Illumina reads to the lambda genome resulted in 100% coverage at 99.3% identity. Thus, two orthogonal sequencing methods were used to establish the *E. coli* and lambda genomic references for assessing the accuracy of in-flight and ground nanopore data. A direct alignment of the 2D reads to the *de novo* assembled genomes showed a median 89% and 86% agreement on base calling for the in-flight and ground data, respectively. Overall, we found that the long, phased genetic data from the MinION is amenable to use in analysis of the sequences generated on the ISS.

### Accuracy and distribution of in-flight nanopore sequencing reads

**Figure 2:**
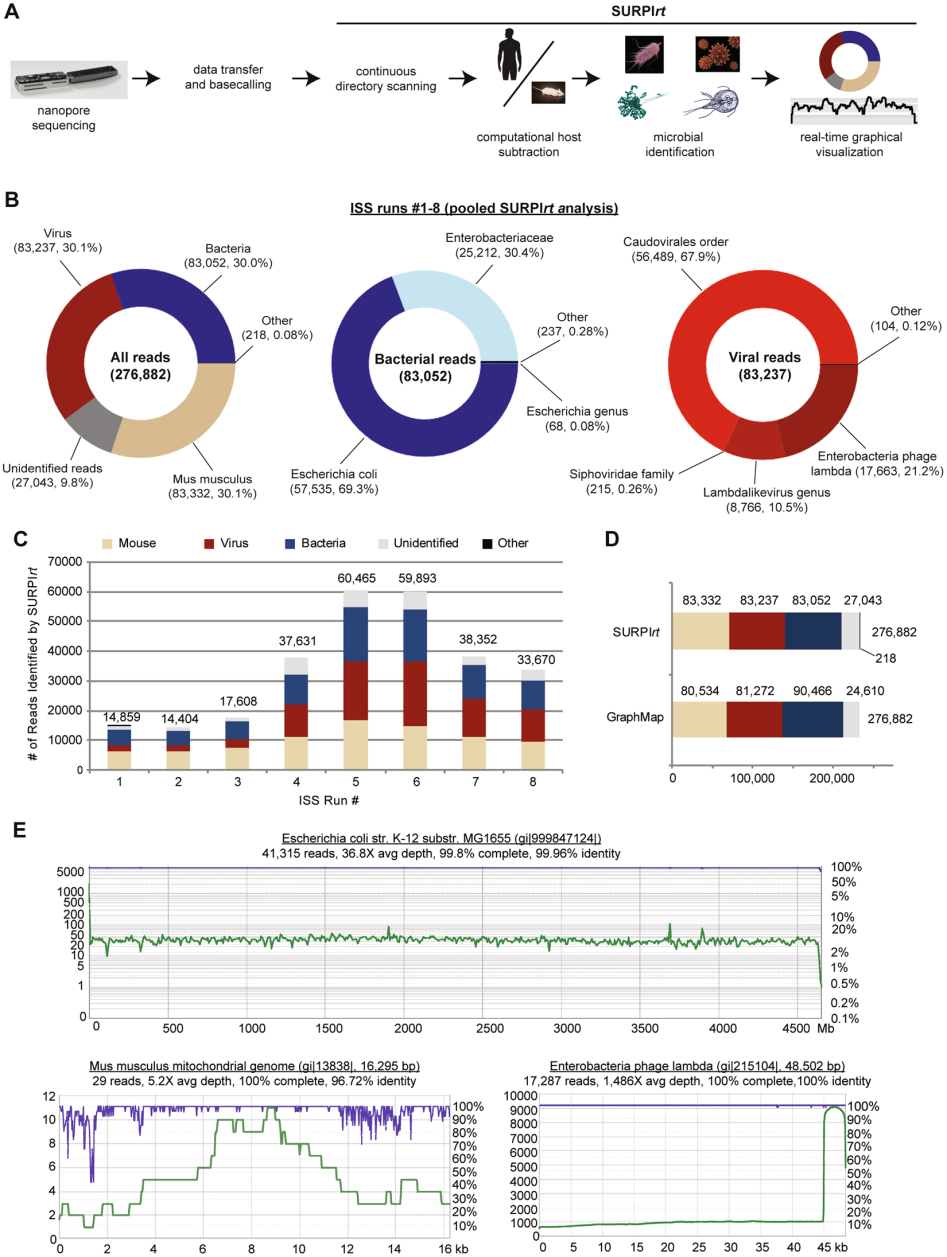
Automated metagenomic analysis of ISS nanopore data. (**A**) Flow chart of the SURPI*rti* bioinformatics pipeline for real-time microbial detection from nanopore data. (**B**) Donut charts of read distributions corresponding to all reads (left), bacteria (middle), and viruses (right) from pooled ISS runs 1 through 8. (**C**) Stacked column plot of reads each from ISS runs 1 through 8 showing distribution of identified organisms. (**D**) Stacked: bar plot of reads from pooled ISS runs 1 through 8 comparing metagenomic detection using SURPI*rt* versus directed alignment using GraphMap for organism identification from nanopore sequencing data.). (**E**) Coverage (green) and pairwise identity plots (purple) of raw nanopore reads mapped to the *E. coli* (upper panel), the mouse mitochondrial (lower left panel), and lambda genomes (lower left panel). Reads are mapped to the most closely matched reference genome identified by SURPI*rt*. Images not generated by the authors were obtained from the CDC Public Health Images Library: human silhouette, image ID 15798, illustrator D. Higgins; giardia, image ID 3394, source A. da Silva and M. Moser; yeast, image ID 300, no attribution possible; virus, image ID 21351, illustrator, A. Eckert; bacteria, image ID 21915, A. Eckert and J. Oosthuizen.

The performance of ISS nanopore sequencing runs 1 through 3 were similar, with total yields of 15,000 to 18,000 reads and a relatively lower proportion of lambda reads relative to *E. coli* and mouse reads (Fig. 1D). Unlike these 6-hour runs, ISS 4 was sequenced for 48 hours, and generated more than twice the number of reads. Interestingly, the percentage of lambda reads generated from ISS run 4 was 30% versus 13% for the first 3 runs, and the relative proportions of lambda, mouse, *E. coli* reads were much closer to the predicted one-third proportions expected from sequencing an equimolar mixture of genomic DNA; similarly, when data obtained from all 8 flow cells were included, 30% of the reads mapped to each organism (Fig. 2A). Across all eight flow cells, approximately 10% of reads (n = 8,960) did not match to either lambda, *E. coli*, or mouse by direct alignment using GraphMap and SURPIrt (Figs 1D and 2A). This was attributed to single-read error rates of 8–20% on the R7 version of flow cells used, as the use of a more sensitive aligner (BLASTn, e-value cutoff = 10^−8^) against the comprehensive NCBI nt database only assigned an additional 2.0% of reads (n = 1,815) as either lambda, *E. coli*, or mouse/human, and failed to detect true sequence matches to other organisms. In contrast, 99.9% of the Illumina reads were properly assigned to one of the 3 organisms (Fig. 1D), although the relative proportions were less uniform than for ISS runs 4–8 (Supplementary Table 2). To gauge the improvement in accuracy of the latest chemistry (R9) for the MinION, we ran an additional equimolar mixture of the three samples on Earth, and we found that the error rates were noticeably lower (5–10%). This updated version of the flow cell uses a different protein nanopore, enabling the higher sequencing accuracy.

### Metagenomic analysis of in-flight nanopore data using the automated SURPIrt pipeline

To demonstrate the feasibility of analyzing sequencing data on the ISS, we ran simulations of real-time metagenomic analysis of all 8 pooled runs from in-flight nanopore data using the automated SURPI*rt* pipeline (Fig. 2A; Supplementary Figure 8)^10,11^. Species-specific reads corresponding to mouse, *E. coli*, and lambda from the sample mixtures were initially identified in SURPI*rt* within 1 minute of beginning sequence analysis by MegaBLAST alignment to the NCBI nt database (word size = 16; e-value = 1 × 10^−5^). The distribution of detected reads could be visualized in real-time as donut charts on a web browser refreshed every 30 s (Fig. 2B).

Automated analyses of all 276,882 nanopore reads in pooled runs 1 through 8 revealed a percent read count distribution of mouse, viral, and bacterial reads of 30.1%, 30.1%, and 30.0%, respectively, consistent with the expected proportions from equimolar mixing of the sample, with only 218 reads (0.08%) mapping to other organisms and 27,043 reads (9.8%) unidentified (Fig. 2B). Taxonomic classification using a lowest common ancestor (LCA) algorithm revealed that nearly all of the bacterial reads (99.7%) mapped to *E. coli* (or its parental taxa), while nearly all of the viral reads (99.9%) mapped to lambda or other phages in the *Caudovirales* order. The proportions of mouse, *E. coli*, and phage reads remained fairly consistent across all 8 runs (Fig. 2C), although relatively fewer lambda reads were detected in the earlier runs 1 through 3.

Overall, the results from the SURPI*rt* pipeline for unbiased pathogen detection were comparable to those obtained by directly mapping the reads to the closest target reference genome in GenBank using GraphMap (Fig. 2D). When individual GraphMap-identified reads were aligned to the reference genome, the mean read lengths and average percent pairwise identities were 6,880 bp and 81.6% for mouse, 5,718 bp and 82.8% for E. coli, and 6,245 bp and 84.1% for phage. The range of read lengths varied considerably from 80 to 72,619 bp (Supplementary Table 2). To generate consensus sequences, individual reads were mapped to the 4.66 Mb E. coli genome, 48.5 kb lambda genome, and a representative mouse sequence, the 16.3 kb mitochondrial genome. Pairwise gapped alignment of the 3 full-length consensus sequences against their corresponding reference genomes using the MAFFT algorithm showed 96.7–100% consensus pairwise identity (Fig. 2E).

### *De novo* genome assembly across the sequencing platforms

To determine if the in-flight nanopore data generated on the ISS could be used for successful *de novo* assembly of the *E. coli* genome, we independently tested two long-read genome assemblers, Miniasm^12^ and Canu^13,14^, using 2D reads pooled from all 9 in-flight sequencing runs. We tested assemblies generated (1) directly from raw 2D data, (2) from remaining reads after background subtraction of mouse sequences, and (3) from reads assigned to *E. coli* only (Supplementary Figure 9). From each of these read subsets, Canu was able to assemble a single contig representing >99% of the complete *E. coli* genome sequence, with ≥98.6% consensus pairwise identity relative to the PacBio reference genome. In contrast, although Miniasm had a significantly faster run time than Canu (<1 minute versus 2 hours on a 64-core server), the assemblies had more contigs, were not as complete (85.1–87.6%), and were less accurate (87.1%). The improved accuracy of Canu relative to Miniasm was also observed previously^15^.

We further tested the ability to run these assemblies not only on computational servers but also on the cloud (the Amazon Elastic Compute Cloud/EC2 platform) and on a laptop. We observed that an 8-core, 32 Gb EC2 instance was sufficient to complete an entire Miniasm assembly within 15 seconds, and a similarly configured laptop with 8 hyperthreaded cores and 64 Gb RAM took less than 40 sec (Supplementary Figure 9). These results on the cloud and laptop showed that real-time genome sequencing and analyses were feasible in space. Analyses of nanopore data were not performed on the ISS in the current study, as available in-flight computing resources were not sufficient (e.g. the on-board Surface Pro 3 tablet uses a single-core i7 processor with 8 Gb of RAM). Nevertheless, in-flight sequence analysis capability could be achieved with the next generation of laptops, supplied to the ISS in June, 2017. As the lambda prophage is inserted into the *E. coli* genome, these data constitute the first *de novo* assemblies of a complete bacterial and viral genome with 100% accuracy, and indeed, the first genome assemblies of any organism from sequence data generated solely off of planet Earth.

## Discussion

Our results of the first-ever DNA sequencing in space indicate that the performance of the MinION sequencing platform was not adversely affected by transport to the ISS, nor by loading or operation in its microgravity environment. Ideally, all experiments on the ground and in-flight would have been performed by the same person; however, we prioritized performing near-simultaneous ground controls with reagents and materials of the same age over having all experiments performed by a single person. Having two separate crew members, K. Rubins and P. Whitson, the latter of whom had never performed any of the sequencing operations prior to her sequencing on the ISS, successfully load samples aboard the ISS reinforces the robustness of the MinION sequencing platform in space. In all cases, the person loading the ground control samples had considerably more experience with the Biomolecule Sequencer payload than the corresponding astronaut. Because sequencer performance was generally better (e.g., more reads obtained) on the ISS despite the more extensive experience of ground team members loading samples, we do not think having different people perform experiments on the ISS and on the ground had an appreciable effect on the quality of sequencing.

Although for this experiment samples were prepared on the Earth, recent simplifications of sample preparation for the MinION sequencer (e.g., Oxford Nanopore Technologies 1D rapid library preparation kits and VolTRAX^TM^ automated sample preparation device) should be straightforward to adapt to the spaceflight environment, and are currently being optimized for deployment to the ISS. Just as on Earth, methods for the extraction of the nucleic acids themselves from ISS-derived samples will need to be tailored for each source. However, the Wetlab-2 project has already successfully demonstrated DNA and RNA extraction aboard the ISS^16^, and microbial DNA extraction could be performed with simple thermal lysis and magnetic bead clean-up, processes that are not gravity-dependent. Furthermore, we recently demonstrated that pipetting using both positive displacement and air displacement pipets was possible on the ISS as well as during microgravity intervals on a parabolic flight^17^.

The data obtained from sequencing aboard the ISS can readily recapitulate the measurements of nucleic acids from phages (lambda), bacteria (*E. coli*), and mammalian (mouse) DNA on Earth. Indeed, across all three species, the base quality and 2D read alignments were routinely above 85%, with equal or superior performance to the identical replicate libraries and flow cells tested on Earth. These results were also true when comparing the skips/base, stays/base, read length, and GC-content of the data. Sequence reads were also validated against sequencing data obtained on PacBio RSII and Illumina MiSeq instruments to confirm their accuracy. *De novo* assembly of nanopore reads collected in-flight enabled the generation of a high-quality genome assembly of *E. coli* (a single full-length contig at ≥98.6% identity). Importantly for microbiome and metagenomic applications, our results demonstrate that a *de novo* assembly of microbial genomes from raw, unfiltered data sequence data corresponding to a complex metagenomic mixture is feasible in space. As with sequence data from any platform, the success of unfiltered assembly from nanopore reads will likely depend on the complexity of the metagenomic background in the sample, depth of sequencing, and error rates, which have been steadily decreasing over time for nanopores^18^. In this study, the individual error rates for identified mouse, *E. coli*, and lambda reads were 18.4%, 17.2%, and 15.9%, respectively, although coverage redundancy during directed or *de novo* assembly, as shown here, can reduce error rates to <2%. We also successfully tested genome assembly of in-flight nanopore reads using a cloud-based platform (Amazon EC2) and laptop. In aggregate, these results clearly validate the implementation of the MinION nanopore sequencer for rapid, *in situ* diagnostics and microbial identification on the ISS, and, ultimately, in any space environment. Furthermore, lightweight sequencing platforms such as the one demonstrated here, coupled with sufficient local computing power, can be directly applied to terrestrial research applications in remote environments. The ability to analyze a subset of samples to assess sampling diversity and quality while in field locations such as the Arctic or on deep-sea drilling expeditions could greatly improve the overall yield of science from these campaigns.

From a spaceflight perspective, in the immediate future the MinION holds the potential to greatly improve the rate at which ISS research can be performed by allowing researchers rapid access to data obtained in-flight, rather than having to wait for sample return. With robust experiment planning and some foresight, research projects that required multiple flights over several years could now be performed in a matter of months, as researchers could monitor experiment progress in real-time and make adjustments as needed (i.e., cadence of time points, identifying a subset of samples that should be returned to Earth for further analysis, etc.). Studies of gene expression in-flight would also be enabled by a sequencing platform on the ISS, and could be performed more robustly and with less risk of experiment failure by reducing the need for storage of labile RNA in a freezer. Analysis aboard the ISS would also serve to help eliminate time constraints, such as those posed by organism re-acclimation upon return to Earth, facilitating more optimal and less arbitrary selection of time points for sample collection and analysis. Nanopore sequencing also has the potential to detect base modifications^19,20^, which could also enable *in situ* epigenetics studies of both DNA and RNA.

As exploration progresses beyond low Earth orbit toward extended missions in *cis*-lunar space and eventually to Mars, changes and advances in nanopore-based sequencing will be needed. Increases in sequencing accuracy will result in improved diagnostic capabilities by reducing the number of false positives and false negatives. As communication delays increase and data transfer rates decrease, local analysis of sequencing data will be essential. Aspects of this challenge are manifested on Earth in remote locations and point-of-care settings of clinical and public health significance, such as “hot spots” from outbreaks due to Ebola or Zika virus^21–23^. In the current study, we used the SURPI*rt* computational platform to simulate an automated metagenomic analysis of nanopore data in real-time, from read processing to microbial identification to genome assembly on both a server and a laptop, and we also showed that rapid (15 sec) assembly was possible, highlighting the ability to use these tools and techniques locally for future missions.

One of the outstanding questions for use of the sequencers such as the MinION in deep space exploration is flow cell stability over the course of an 18 to 36 month mission. Extreme temperatures and increased galactic and cosmic radiation exposure are less of a concern for flow cell stability during human missions, as crew members will require shielding from these conditions as well^24^. For robotic missions, however, enhancements in flow cell stability will likely be needed, which could be achieved through the development of more robust membranes in which the pores are embedded or with improvements in the resolving power of solid state nanopores. It is worth noting however, that the present work has demonstrated flow cells are stable after 6 months in orbit, which is on par with at least a one-way transit to Mars; and radiation exposure would not seem to be a significant factor for protein nanopore stability: the Curiosity rover measured <1 Gray of radiation during its transit to Mars and 0.18 to 0.225 mGray per day on the Martian surface^25,26^, which are radiation doses orders of magnitude lower than doses (3,500 Gray) that proteins have been demonstrated to tolerate with no loss in function^27^. Flow cell reusability would be of tremendous benefit on Mars and other deep-space missions due to stringent mass limits imposed by cost and propulsion constraints; we were able to demonstrate in a limited fashion the reusability of flow cells aboard the ISS, where a minor decrease in the number of available pores was observed between the first and second flow cell loadings, and overall data yields were comparable to those obtained from 48 hour sequencing runs on flow cells only used once. Assuming sufficient flow stability is achieved, nucleic acid sequencing could play an important role on crewed missions to Mars to monitor crew health.

Once on Mars or another planetary surface, the sequencing platform would then become a powerful tool for surveillance and exploration. DNA-based life could be rapidly detected, enabling identification of Earth-derived contamination and perhaps even characterization of indigenous Martian life if it also uses DNA. Beyond life as we know it, direct analysis of molecules by nanopores has been used to detect base modifications in DNA^19,20^, to identify pathogens in clinical samples^11^, to sequence RNA^23,28,29^, and even to characterize proteins^30^. The ability of nanopore analyzers to accommodate a range of polymers increases the chance of detecting extraterrestrial life, which could use different bases or sugars in its genetic material beyond canonical nucleotide-based DNA and RNA.

## Materials and Methods

### Code availability

The Megablast-based SURPI*rt* code for metagenomic analysis of the data generated from this study can be found on the Github repository for the UCSF Chiu laboratory at https://github.com/chiulab. The remaining scripts are available at https://pbtech-vc.med.cornell.edu/git/mason-lab/nanopore_in_space/tree/master.

### Data availability

The datasets generated or analyzed during the current study are available from the authors on reasonable request; base-called.fastq files are available in the NASA GeneLab database under accession number 84; https://genelab-data.ndc.nasa.gov/genelab/accession/GLDS-84.

### Spaceflight Hardware

The full payload included the following items: two MinION devices, a USB 3.0 cable, nine R7.3 flow cells (Oxford Nanopore Technologies), nine sample syringes containing ground-prepared genomic DNA, nine empty sample syringes for air bubble removal, and a configuration flow cell (Supplementary Figure 10). A pipette kit including 10, 100, and 1,000 μl Rainin positive displacement pipettes (Mettler Toledo, Oakland, CA) and associated tips was included for contingency purposes if the syringe was not sufficient for bubble removal. These items were all launched from Cape Canaveral Air Force Base on the SpaceX CRS-9 Dragon capsule on July 18th, 2016. The MinKNOW™ software (Oxford Nanopore Technologies) required for operation of the MinION was loaded on a Microsoft Surface Pro 3 tablet and delivered to the ISS on the Orbital ATK OA-6 Cygnus Space Station Resupply Vehicle, which launched on March 22, 2016.

### Library Preparation and Sequencing

Samples analyzed in this study contained sequence libraries prepared from mixtures of female mouse BALB/C (Zyagen, San Diego, CA), *Escherichia coli* K-12 (Zyagen), and *N* ^6^-methyladenine-free bacteriophage lambda (New England Biolabs, Ipswich, MA) genomic DNA. These species were chosen based on their being model organisms corresponding to a eukaryote (mammal), bacterium, and virus, respectively. The samples were aliquoted for library preparation on three platforms: Oxford Nanopore Technologies (ONT) MinION (v6), Illumina MiSeq (v2), and the Pacific Biosciences RSII.

### MinION Library Preparation and Sequencing (Ground and ISS)

Aliquots of DNA for mouse, *E. coli*, and lambda libraries were sheared individually using Covaris g-TUBEs (Covaris, Boston, MA) by centrifugation at 4,800 × *g* for 2 min to produce fragments that were predominantly 8 kb. Fragmentation was verified using a 2100 Agilent Bioanalyzer (Agilent Technologies, Santa Clara, CA). Sheared mouse, *E. coli*, and lambda DNA samples were quantified using a Qubit 2.0 fluorometer (Thermo Fisher Scientific, Waltham, MA) and combined in equal abundances, targeting 1.5 µg total (0.5 µg each). Mixed DNA samples then underwent treatment to repair residual nicks, gaps, and blocked 3′ ends using Formalin-Fixed, Paraffin-Embedded (FFPE) DNA Repair Mix (New England Biolabs), with modifications to the manufacturer’s protocol to include a 0.5X Agencourt AMPure XP system magnetic bead clean-up (Beckman Coulter Genomics, Brea, CA) to remove small fragments of DNA. The repaired DNA was then prepared for sequencing according to Oxford Nanopore Technologies’ procedure for the SQK-MAP-006 Kit. Following library preparation, individual samples were mixed with a ~100 ng aliquot of pre-sequencing library (150 µl) with 162.5 µl of 2X running buffer, 6.5 µl fuel mix (Oxford Nanopore Technologies) and 156 µl molecular-grade sterile deionized water to a total volume of 450 µl. This volume was loaded into a 1 ml syringe and the syringe was capped. A total of 18 samples were prepared: nine flight samples and nine ground control samples. Capped syringes containing the samples were packaged with an identical 1 ml syringe for potential bubble removal and syringe tips were placed inside of a large plastic tube to facilitate transport. After syringe loading, the tubes were stored at −80 °C.

### Future flight-compatible sample preparation

While the DNA sequenced in-flight was prepared on the ground, recent demonstrations of the transfer of microliter fluid volumes with conventional pipettes in a microgravity environment^17,19^, in combination with Oxford Nanopore Technologies commercially-available rapid library preparation kit, lend themselves to the reality of sample preparation on the ISS. The miniPCR system is already onboard the ISS and has been used as both a heat block and thermal cycler in ground-based testing to support rapid 1D library preparation and amplifications of low input samples, respectively. Although a manual sample preparation process could be implemented on the ISS in the near future to enable the sequencing of spaceflight samples, flight certification of the soon-to-be-released sample and library preparation device, VolTRAX™ by Oxford Nanopore Technologies, could fully automate the entire process.

### Ground Processing of Cold Stowage Hardware

The nine flight samples were removed from the −80 °C freezer, placed on dry ice in a Styrofoam cooler, and shipped overnight to the launch processing facility at Kennedy Space Center (KSC). Once at KSC, the samples were maintained in a −80 °C freezer until transfer to the powered freezer on the SpaceX Dragon Capsule. To more closely approximate the handling of the flight samples, the nine ground control samples were also removed from the freezer, placed on dry ice in a Styrofoam cooler and allowed to sit on the laboratory bench for the same amount the time that the flight samples were out of the freezer during transit. A total of 18 R7.3 flow cells from the same manufacturing lot were shipped directly from the manufacturer to KSC for flight (9) and JSC to serve as ground controls (9). Upon receipt, the flow cells were stored at +4 °C until the time of launch. The flow cells and samples were maintained within the launch processing labs at KSC until 48 h before the launch, at which point they were loaded onto the vehicle.

### Launch and ISS Stowage Conditions

Flow cells were launched in a double cold bag in which the temperature was maintained between +2 and +8 °C. The DNA samples were launched in a powered freezer in which temperatures were maintained between −80 and −90 °C. Upon docking with the ISS, the flow cells and DNA samples were transferred to refrigerator (+2 to +8 °C) and freezer (−80 to −90 °C) dewars within the Minus Eighty Degree Laboratory Freezer for ISS (MELFI), respectively. All other items were stored at ambient ISS conditions.

### Sequencing Experiments

Following complete charging of the Surface Pro 3, it was connected to the MinION via USB cable. Prior to the first sequencing experiment, a configuration test cell was inserted into the MinION device for the configuration test MAP_CTC_Run.py in the MinKNOW software version 0.51.1.39 b201511042121 to ensure proper data exchange between the MinION device and the Surface Pro 3. This version of MinKNOW software was modified so that internet connectivity was not required. To initiate the experiment, a flow cell and DNA sample syringe tube were collected from their respective MELFI dewars and allowed to equilibrate to ambient temperature (10–60 min). The flow cell was inserted into the sequencing device and the bubble removal syringe was used to remove the air immediately adjacent to the sample loading port (~15 μl of air). Flow cell priming was performed by loading approximately 250 μl of the sample containing fuel mix and running buffer and allowing 10 min to elapse prior to loading the remaining 250 μl of sample. Sequencing was initiated using MAP_Lambda_Burn_In_Run_SQK_MAP006.py protocol selected from the MinKNOW software. These procedures were optimized based on our parabolic flight experience^9^ in the following ways: prepared samples were stored at −80 °C or below until use rather than −20 °C; we also added headspace at the back of the sample syringe prior to freezing to simplify loading procedures for the crew members; half the sample was used to flush the flow cell, followed by a 10 minute wait, and loading of the remainder of the sample rather than loading the sample as a single 450 μL volume; and the sequencing run was not initiated until after sample loading was complete, giving the flow cell sufficient time to reach ambient temperature. Real-time communications with the ISS allowed the ground control flow cells and samples to be retrieved, temperatures to equilibrate, and samples to be injected for priming and final sample loading in a synchronous manner. For the flow cell reuse experiment (6.1 and 6.2), a sample library was loaded as described above and a 6 hour sequencing was initiated. After 6 hours, the flow cell was removed from the MinION and stored overnight (~18 hours) at 4 C. The flow cell was retrieved and placed into the MinION and a second sample thawed, where it was loaded in two stages, after which a 48 hour sequencing run was initiated.

### PacBio RSII Library Preparation and Sequencing

Single Molecule, Real-Time (SMRT) sequencing libraries were prepared using the SMRTbell Template Prep Kit 1.0 (Pacific Biosciences) and 20 kb Template Preparation Using BluePippin Size-Selection System protocol (Pacific Biosciences). For each sample, 5 μg were used. Library quality and quantity were determined using an Agilent 2200 TapeStation and Qubit dsDNA BR Assay (Life Technologies), respectively. Sequencing was conducted using P6-C4 chemistry and a v3 SMRT Cell (Pacific Biosciences) at Weill Cornell Medicine.

### Illumina MiSeq Library Preparation and Sequencing

Sequencing libraries were prepared from 1 ng of sample using the NexteraXT kit (Illumina) according to the manufacturer’s protocol. Libraries were indexed with dual 8-nt barcodes on each end of the sequencing amplicon. In total, 4 dual-indexed DNA sequencing libraries were constructed, corresponding to lambda bacteriophage, *E. coli*, mouse, and an equimolar mixture of DNA from the 3 organisms. Libraries were quantitated using the Agilent Bioanalyzer and Qubit spectrophotometer and sequenced on an Illumina MiSeq as a 1 × 160 bp single-end sequencing run. The approximate percentage of the run allocated to each library as determined by the quantified input concentration was 3% for lambda bacteriophage, 17% for *E. coli*, 30% for mouse, and 50% for the equimolar mixture.

### Basecalling and Read Selection

Basecalling was performed using the Metrichor workflow “2D Basecalling for SQK-MAP006 - v1.107”. We used a custom shell script to extract one read from each fast5 file for further quantification and analysis, selecting the 2D read where available, and the higher quality of the 1D template or complement read where not.

### GraphMap and Calculation of Species Counts/Proportions

As described previously^9^, we aligned to a combined *Enterobacteria lambda phage* (NCBI reference sequence NC_001416.1), *Escherichia coli* (NCBI reference sequence NC_000913.3), and *Mus musculus* (mm10, GRCm38.p4) genome using GraphMap version 0.3.0, with the command “graphmap align -r $ref -d $fi -o $name.sam”, which saves the top result for each read. We used the results to count the number of reads mapping to each of the three species and the fraction identity between reads and references.

For comparison of the relative species proportions in the sample mixture between the nanopore in-flight runs and the Illumina data, we separately aligned the Illumina and nanopore reads to the *Mus musculus, E. coli*, and lambda phage genomes using Bowtie2 in local alignment mode at default settings^31^ and GraphMap^32^, respectively. We then ensured only one unique mapping per read, which include assigning all lambda reads to the lambda genome (as the complete lambda genome is integrated in the *E. coli* chromosome), prior to calculating relative species proportions. Individual reads were mapped to the corresponding reference genome using the Geneious software package version 8.1.9 (Biomatters, Inc.). After determination of the consensus sequence in Geneious, consensus pairwise identities were calculated by taking each consensus sequence and performing a pairwise gapped alignment using the MAFFT algorithm (v7.0)^33^ at default settings (algorithm = “Auto”; scoring matrix = “200PAM/k = 2”, gap open penalty = 1.53; offset value = 0.123), as implemented in the Geneious software package.

### *De novo* genome assembly from PacBio and Illumina data

To ensure that our *E. coli* alignments and sequencing measures were not a result of any strain or sample-specific genetic drift or contamination, we performed a *de novo* assembly of the *E. coli* genome used in this study using the PacBio data. We used the Hierarchical Genome Assembly Process (HGAP, v2) for read-cleaning and adapter trimming (pre-assembly), *de novo* assembly with Celera Assembler, and assembly polishing with Quiver^34^. Raw sequencing reads were filtered for length and quality such that the minimum polymerase read score was 0.8, the minimum subread length was 500 bp, and the minimum polymerase read score was greater than 100. The assembly was generated using CeleraAssembler v1 with the default parameters and was polished using the Quiver algorithm^34^. Our assembly yielded a single contig of 4,734,145 base pairs at >99.7% accuracy and confirmed the *E. coli* sample as strain K-12.

We next performed independent *de novo* and directed assemblies of the *E. coli* genome using single-end Illumina data. Raw Illumina reads were preprocessed for trimming of adapters and removal of low-complexity and low-quality sequences. *E. coli* reads were identified using Bowtie2 alignment against the *E. coli* reference genome in local alignment mode at default parameters. *De novo* assembly was performed using the SPAdes genome assembly v3.8.2^35^ with the “careful” parameter. The *de novo* assembled contigs as well as the original dataset of Illumina reads were then separately mapped to the PacBio *E. coli* genome assembly using Geneious v8.0 (Biomatters, Inc.). Illumina reads aligning to lambda by Bowtie2 were also mapped to the lambda phage genome (LAMCG) using Geneious.

### SURPIrt (sequence-based ultra-rapid pathogen identification, real-time) analysis

The SURPI*rt* pipeline is a real-time analysis pipeline for automated metagenomic pathogen detection and reference-based genomic assembly from nanopore sequencing data. Modeled after previously published SURPI^10^ and Metapore^11^ software, SURPI*rt* incorporates Linux shell scripts and code from the Python, Perl, Javascript, HTML, and Go programming languages. SURPI*rt* is currently being developed for analysis of clinical human samples, but was customized here for automated analysis of the NASA test mixture of mouse, *E. coli*, and lambda bacteriophage DNA. Specifically, reads are aligned successively using Megablast^36^ to mouse, viral RefSeq.^37^, bacterial RefSeq.^37^, and non-chordate eukaryotic^10^ sequence databases for detection of host (mouse) and microbial reads (Supplementary Figure 11). Reads are then taxonomically classified using a lowest common ancestor algorithm^38^, and graphical results are provided as tables, pie charts, and coverage maps. SURPI*rt* can be run on a server, cloud, or laptop.

We simulated a real-time SURPI*rt* analysis of the NASA runs using both ground and in-flight nanopore data downloaded from the ISS (Supplementary Figure 11). In-flight analyses were not possible as a laptop with the necessary computational power and a local basecaller, obviating the requirement for an Internet connection, were not available at the time of these studies. For the simulation, the SURPI*rt* pipeline was run in automated mode to continually scan a download directory containing raw FAST5/HDF files from the MinION sequencer for batch analysis. For a preset number of reads per batch (n = 200 for this analysis) corresponding to ~2 minutes of elapsed time, the 2D read or either the template or complement read, depending on which is of higher quality, was extracted from each FAST5 file, and a FASTQ file generated using HDF5 Tools (ref: HDF5/Tools API Specification, http://www.hdfgroup.org/HDF5/doc/RM/Tools.html, accessed September 18th, 2016). Reads corresponding to mammalian reads (i.e., mouse) were subtracted computationally using MegaBLAST (word size 16, e-value cutoff = 10^−5^) alignment to the *M. musculus* reference genome. Remaining reads were then aligned consecutively to a viral database (NCBI Viral RefSeq), bacterial database (NCBI Bacterial RefSeq), and to a non-chordate eukaryotic database (extracted from NCBI GenBank NT). For each read, the single best match by e-value was retained, and the corresponding NCBI Genbank identifier (gi) assigned to the best match (“top hit”) was then annotated by taxonomic lookup of the corresponding lineage, family, genus, and species. For each microbial species detected, the top hit was chosen to be the reference gi in the NCBI nt database (among all reference sequences assigned to that species) with the highest number of aligned reads, with priority given to reference sequences in the following order: (1) complete genomes, (2) complete sequences, or (3) partial sequences or individual genes. In the case of multiple top hits, reads were taxonomically classified according to the lowest common ancestor algorithm^11^. For automated real-time results visualization^11^, a live taxonomic count table was generated by SURPI*rt* and displayed as a donut chart using the CanvasJS graphics suite (http://canvasjs.com/, accessed September 26^th^, 2016) with the chart refreshing every 30 s.

### *De novo* genome assembly from nanopore data

We used Minimap v0.2-r124-dirty and Miniasm v0.2-r137-dirty as described in the github tutorial, using 8 threads (https://github.com/lh3/miniasm). Threeo assemblies were generated, (1) using 2D reads that mapped exclusively to *Escherichia coli* K12 MG1655 using GraphMap from the four runs on the ISS, and (2) using all 2D reads from the ISS that did not map to either human or mouse (using the hg38 human and mm10 mouse reference genomes), and (3) using all raw 2D reads. In parallel, we used Canu v1.73^13^ at default parameters using a specified target genome size of 4.8 MB for *de novo* assembly. Runs were performed using both a 64-core computational server with 512 gigabytes (Gb) memory and an 8-hyperthreaded core laptop with 64 Gb memory. Three assemblies were generated using the same read sets as for Miniasm. Assembly metrics, including N50, were calculated by the “abyss-stats.pl” program in ABySS^39^. The assembled contigs were mapped to the PacBio-generated *E. coli* genome and visualized using Mauve version 2.4.0^40^. Consensus pairwise identities of the *de novo*-assembled *E. coli* genomes were estimated using JSpeciesWS^41^ after specifying the use of MUMmer^42^ for pairwise identity calculations.

## Electronic supplementary material

Supplementary Information

## References

[1] Taylor GR, Konstantinova I, Sonnenfeld G, Jennings R (1997). Changes in the immune system during and after spaceflight. Adv Space Biol Med.

[2] Wilson JW (2007). Space flight alters bacterial gene expression and virulence and reveals a role for global regulator Hfq. Proc Natl Acad Sci USA.

[3] Cho I, Blaser MJ (2012). The Human Microbiome: at the interface of health and disease. Nature reviews. Genetics.

[4] Kinross JM, von Roon AC, Holmes E, Darzi A, Nicholson JK (2008). The human gut microbiome: implications for future health care. Current gastroenterology reports.

[5] Johnson SS, Zaikova E, Goerlitz DS, Bai Y, Tighe SW (2017). Real-Time DNA Sequencing in the Antarctic Dry Valleys Using the Oxford Nanopore Sequencer. Journal of Biomolecular Techniques: JBT.

[6] Edwards, A. *et al*. Deep Sequencing: Intra-Terrestrial Metagenomics Illustrates The Potential Of Off-Grid Nanopore DNA Sequencing. *bioRxiv* **133413**, 10.1101/133413 (2017).

[7] Hoenen T (2016). Nanopore sequencing as a rapidly deployable Ebola outbreak tool. Emerging infectious diseases.

[8] Quick J (2016). Real-time, portable genome sequencing for Ebola surveillance. Nature.

[9] McIntyre AB (2016). Nanopore sequencing in microgravity. npj Microgravity.

[10] Naccache SN (2014). A cloud-compatible bioinformatics pipeline for ultrarapid pathogen identification from next-generation sequencing of clinical samples. Genome research.

[11] Greninger AL (2015). Rapid metagenomic identification of viral pathogens in clinical samples by real-time nanopore sequencing analysis. Genome medicine.

[12] Li, H. Minimap: Experimental tool to find approximate mapping positions between long sequences https://github.com/lh3/minimap/ (2015).

[13] Koren, S. *et al*. Canu: scalable and accurate long-read assembly via adaptive k-mer weighting and repeat separation. *bioRxiv*; 10.1101/071282 (2017). 10.1101/gr.215087.116 PMC5411767 28298431

[14] Koren, S., Walenz, B. P., K, B., Miller, J. R. & Phillippy, A. M. Canu: scalable and accurate long-read asembly via adaptive k-mer weighting and repeat separation. *bioRxiv*, 10.1101/071282 (2016). 10.1101/gr.215087.116 PMC5411767 28298431

[15] Judge, K. *et al*. Comparison of bacterial genome assembly software for MinION data and their applicability to medical microbiology. *Microbial Genomics* **2**; 10.1099/mgen.0.000085 (2016). 10.1099/mgen.0.000085 PMC5320651 28348876

[16] Parra M (2017). Microgravity validation of a novel system for RNA isolation and multiplex quantitative real time PCR analysis of gene expression on the International Space Station. PLOS ONE.

[17] Rizzardi LF (2016). Evaluation of techniques for performing cellular isolation and preservation during microgravity conditions. Npj Microgravity.

[18] Goodwin S, McPherson JD, McCombie WR (2016). Coming of age: ten years of next-generation sequencing technologies. Nat Rev Genet.

[19] Rand AC (2017). Mapping DNA methylation with high-throughput nanopore sequencing. nature methods.

[20] Simpson JT (2017). Detecting DNA cytosine methylation using nanopore sequencing. nature methods.

[21] Quick J (2016). Real-time, portable genome sequencing for Ebola surveillance. Nature.

[22] Sardi SI (2016). Coinfections of Zika and Chikungunya viruses in Bahia, Brazil, identified by metagenomic next-generation sequencing. Journal of clinical microbiology.

[23] Kilianski A (2016). Use of Unamplified RNA/cDNA–Hybrid Nanopore Sequencing for Rapid Detection and Characterization of RNA Viruses. Emerging infectious diseases.

[24] Cucinotta FA (2001). Space radiation cancer risks and uncertainties for Mars missions. Radiation research.

[25] Hassler DM (2013). Mars’ surface radiation environment measured with the Mars Science Laboratory’s Curiosity rover. Science.

[26] Zeitlin C (2013). Measurements of energetic particle radiation in transit to Mars on the Mars Science Laboratory. Science.

[27] Ruhl S (2011). Integrity of proteins in human saliva after sterilization by gamma irradiation. Appl. Environ. Microbiol.

[28] Smith, A. M., Jain, M., Mulroney, L., Garalde, D. R. & Akeson, M. Reading canonical and modified nucleotides in 16S ribosomal RNA using nanopore direct RNA sequencing. *bioRxiv*, **132274**; 10.1101/132274 (2017).

[29] Garalde, D. R. *et al*. Highly parallel direct RNA sequencing on an array of nanopores. *bioRxiv* **068809**, 10.1101/068809 (2016). 10.1038/nmeth.4577 29334379

[30] Nivala, J., Marks, D. B. & Akeson, M. Unfoldase-mediated protein translocation through an alpha-hemolysin nanopore. *Nat Biotechnol* **31**, 247–250, doi: 1038/nbt.2503 (2013). 10.1038/nbt.2503 PMC3772521 23376966

[31] Langmead B, Salzberg SL (2012). Fast gapped-read alignment with Bowtie 2. Nat Methods.

[32] Sovic I (2016). Fast and sensitive mapping of nanopore sequencing reads with GraphMap. Nature communications.

[33] Katoh K, Standley DM (2013). MAFFT Multiple Sequence Alignment Software Version 7: Improvements in Performance and Usability. Molecular Biology and Evolution.

[34] Chin CS (2013). Nonhybrid, finished microbial genome assemblies from long-read SMRT sequencing data. Nat Methods.

[35] Bankevich A (2012). SPAdes: a new genome assembly algorithm and its applications to single-cell sequencing. J Comput Biol.

[36] Altschul SF, Gish W, Miller W, Myers EW, Lipman DJ (1990). Basic local alignment search tool. J Mol Biol.

[37] Pruitt KD, Tatusova T, Maglott DR (2007). NCBI reference sequences (RefSeq): a curated non-redundant sequence database of genomes, transcripts and proteins. Nucleic Acids Research.

[38] Huson DH, Auch AF, Qi J, Schuster SC (2007). MEGAN analysis of metagenomic data. Genome Research.

[39] Simpson JT (2009). ABySS: a parallel assembler for short read sequence data. Genome Res.

[40] Darling AE, Mau B, Perna N (2010). T. progressiveMauve: multiple genome alignment with gene gain, loss and rearrangement. PLoS One.

[41] Richter M, Rossello-Mora R, Oliver Glockner F, Peplies J (2016). JSpeciesWS: a web server for prokaryotic species circumscription based on pairwise genome comparison. Bioinformatics.

[42] Kurtz S (2004). Versatile and open software for comparing large genomes. Genome Biol.

