# Supplementary material for: Nanopore DNA Sequencing and Genome Assembly on the International Space Station
Source: Sci Rep. 2017 Dec 21;7:18022. doi: 10.1038/s41598-017-18364-0 (PMC5740133; doi:10.1038/s41598-017-18364-0)
Supplement: Supplementary file 1 — Supplementary Information [file 41598_2017_18364_MOESM1_ESM.pdf]

Nanopore DNA Sequencing and Genome Assembly on the International Space Station

Sarah L. Castro-Wallace[1], Charles Y. Chiu[2,3], Kristen K. John[4], Sarah E. Stahl[5], Kathleen H. Rubins[6], Alexa B.R. McIntyre[7], Jason P. Dworkin[8], Mark L. Lupisella[9], David J. Smith[10], Douglas J. Botkin[11], Timothy A. Stephenson[12], Sissel Juul[13], Daniel J. Turner[13], Fernando Izquierdo[13], Scot Federman[2,3], Doug Stryke[2,3], Sneha Somasekar[2,3], Noah Alexander[7], Guixia Yu[2,3], Christopher E. Mason[7,14,15], and Aaron S. Burton[16*]

Supplementary Information

Supplementary Table 1. Comparison of flight and ground sequencing run statistics

Run (Experimenter)	Date	Sample ID	Library input (ng)	Run duration (hours)	Pre-flight pores from platform QC	Total active pores after sample loading (distribution)	Total raw reads
G1 (Stahl)	August 26th, 2016	1	102	6	1,375	640 (365, 193, 68, 14)	14,932
G2 (Stahl)	September 3rd, 2016	1	102	6	1,121	188 (188, 25, 2, 0)	778
G3 (Stahl)	September 7th, 2016	1	102	6	1,089	742 (404, 232, 87, 19)	16,846
G4 (Burton)	September 13th, 2016	4	99	48	1,548	1432 (506, 445, 331, 150)	18,836
G5 (Stahl)	October 18th, 2016	2	96	48	1,137	363 (279, 73, 11, 0)	15,265
G6 (injection 1; Stahl))	October 25th, 2016	3	105	6	1,409	361 (253, 90, 17, 1)	4,981
G6 (injection 2; Stahl)	October 26th, 2016	3	105	48	1,409[a]	172 (132, 39, 1, 0)[b]	616
G7 (Stahl)	November 26th, 2016	2	96	18[c]	1,039	796 (429, 248, 87, 22)	43,047
G8 (Stahl)	January 9th, 2017	2	96	48	991	717 (382, 233, 81, 21)	15,252
				**Average**	**1,214**	**655**	**14,506**
						Total reads	130,553
ISS1 (Rubins)	August 26th, 2016	1	102	6	969	727 (394, 231, 87, 15)	14,903
ISS2 (Rubins)	September 3rd, 2016	1	102	6	1,148	1014 (439, 322, 199, 54)	16,931
ISS3 (Rubins)	September 7th, 2016	1	102	6	1,313	1066 (456, 364, 189, 57)	17,715
ISS4 (Rubins)	September 13th, 2016	4	99	48	1,081	880 (408, 289, 144, 41)	40,144
ISS5 (Rubins)	October 18th, 2016	2	96	48	897	702 (376, 214, 97, 15)	60,864
ISS6 (injection 1; Rubins)	October 25th, 2016	3	105	6	1,067	886 (443, 284, 122, 37)	18,604
ISS6 (injection 2; Rubins)	October 26th, 2016	3	105	48	1,067[a]	699 (384, 206, 86, 23)[b]	41,973
ISS7 (Whitson)	November 26th, 2016	2	96	42[c]	1,055	951 (452, 318, 146, 35)	39,154
ISS8 (Whitson)	January 9th 2017	2	96	48	1,220	924 (422,297,159,46)	34,026
				**Average**	**1,094**	**894**	**31,590**
						Total reads	284,314

[a]The same flow cell was used for 6.1 and 6.2 so the platform QC numbers are the same.
[b]The number of active pores from 6.2 was not included in the average number of pores across all flow cells.
[c]Denotes sequencing runs that terminated early due to the Surface Pro 3 running out of power.

Supplementary Table 2. Statistics for mouse, *E. coli*, and lambda phage reads identified using GraphMap

		# reads	average % pairwise identity	mean length (bp)	range of lengths (bp)
mouse	ISS Flight 1	5,941	82.90%	6,018	[153 - 41,291]
	ISS Flight 2	5,809	84.60%	6,259	[153 - 30,149]
	ISS Flight 3	7,111	84.90%	6,248	[224 - 37,378]
	ISS Flight 4	11,061	79.00%	6,135	[224 - 28,178]
	ISS Flight 5	16,478	79.40%	7,210	[94 - 47,821]
	ISS Flight 6	14,497	80.50%	6,969	]152 - 46,537]
	ISS Flight 7	10,486	83.00%	7,379	[80 - 55,294]
	ISS Flight 8	9,151	83.70%	7,917	[106 - 47,754]
	TOTAL	80,534	81.6% [+/- 7.7%]	6,880	[80 - 55,294]
E. coli	ISS Flight 1	1,884	84.20%	6,015	[343 - 39,907]
	ISS Flight 2	1,864	86.00%	6,419	[181 - 48,086]
	ISS Flight 3	2,312	85.90%	6,341	[209 - 31,226]
	ISS Flight 4	11,077	81.40%	4,348	[160 - 51,783]
	ISS Flight 5	19,553	81.20%	5,981	[190 - 72,619]
	ISS Flight 6	21,546	82.00%	5,450	[152 - 64,359]
	ISS Flight 7	12,611	84.40%	6,083	[177 - 53,327]
	ISS Flight 8	10,425	85.30%	6,474	[125 - 57,043]
	TOTAL	81,272	82.8% [+/- 7.4%]	5,718	[125 - 72,619]
lambda phage	ISS Flight 1	5,497	84.30%	5,961	[165 - 29,732]
	ISS Flight 2	5,404	86.50%	6,304	[188 - 39,327]
	ISS Flight 3	6,575	86.50%	6,202	[157 - 32,341]
	ISS Flight 4	11,007	81.60%	5,951	[133 - 28,442]
	ISS Flight 5	19,718	82.60%	6,291	[153 - 39,871]
	ISS Flight 6	19,168	83.50%	6,230	[149 - 38,605]
	ISS Flight 7	12,368	85.60%	6,358	[133 - 31,445]
	ISS Flight 8	10,729	86.00%	6,502	[133 - 39,190]
	TOTAL	90,466	84.1% [+/- 7.2%]	6,245	[133 - 39,871]

Supplementary Figures and Legends

Supplementary Figure 1. Metrichor/Epi2me analysis of Earth and MinION reads 1 – 4. Oxford Nanopore Technologies created a custom chained workflow consisting of 1D basecalling of the raw fast5 files, then 2D basecalling, extraction of quality score and read-length information, and finally read alignment. The workflow is capable of processing individual reads as soon as they are generated on the MinION, meaning that data can be analysed in almost real-time. Due to internet limitations on the ISS, data was downloaded and processed immediately on Earth following completion of each run. In this way, basecalling and alignment of the data were performed almost simultaneously, allowing the success of the experiment to be confirmed very shortly after the workflow was started. For alignment, the workflow first takes 2D reads and uses Minimap [1] to establish whether each read maps to the mouse BALB/C, E. coli K-12 or lambda phage genomes. When reads are found to align to both lambda and *E. coli* genomes, the workflow uses BLAST [2] to identify the correct placement. Any reads that still cannot be resolved in this way are placed into the 'unknown' group. Reads that do not align to any of the three reference genomes are placed into the no_match group (Supplementary Figures 1a and 1b). Supplementary Figure 1c shows read counts for two Earth and all four ISS runs together; data for G2 were not included.

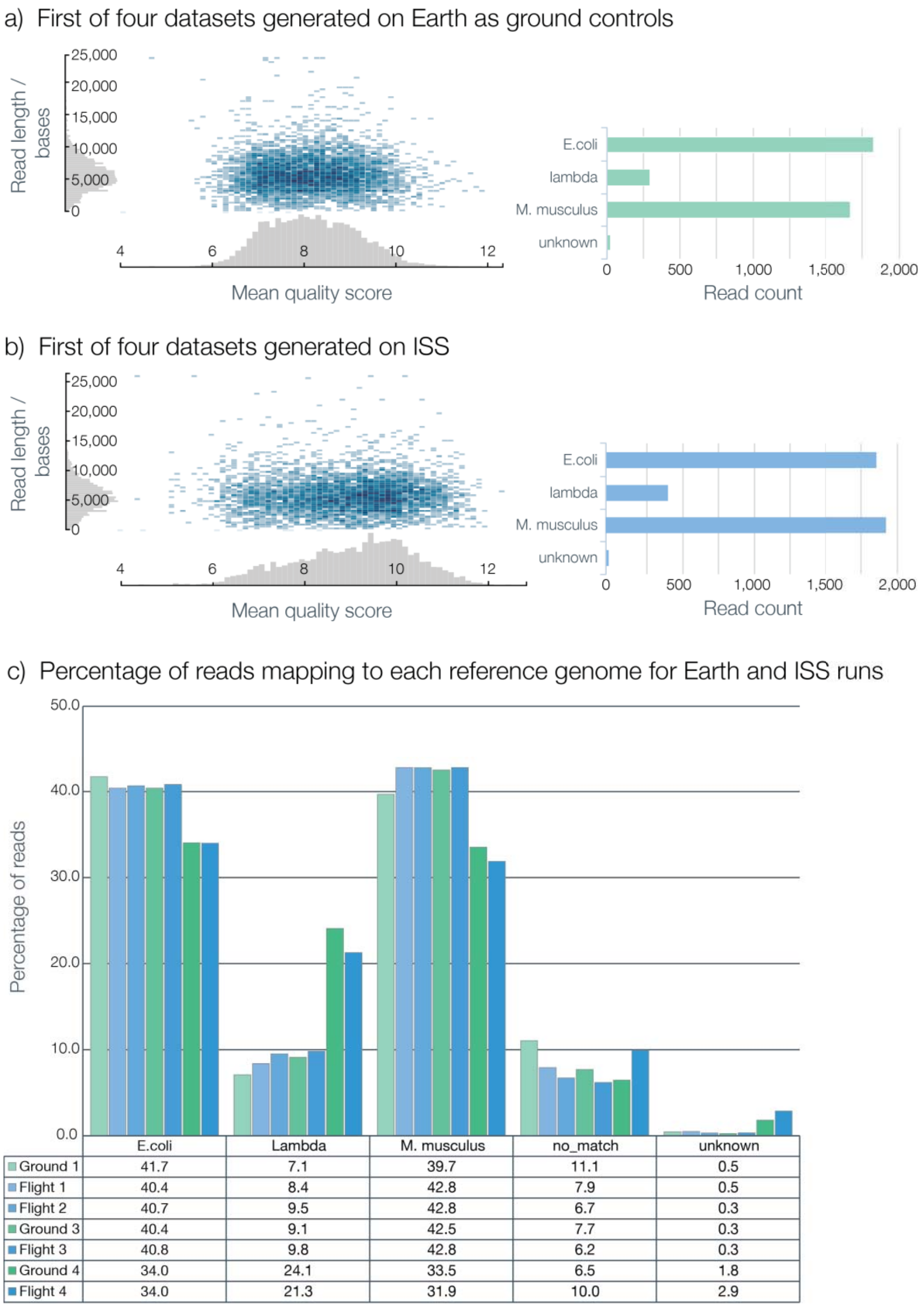

	E.coli	Lambda	M. musculus	no_match	unknown
Ground 1	41.7	7.1	39.7	11.1	0.5
Flight 1	40.4	8.4	42.8	7.9	0.5
Flight 2	40.7	9.5	42.8	6.7	0.3
Ground 3	40.4	9.1	42.5	7.7	0.3
Flight 3	40.8	9.8	42.8	6.2	0.3
Ground 4	34.0	24.1	33.5	6.5	1.8
Flight 4	34.0	21.3	31.9	10.0	2.9

Supplementary Figure 2. Quality metrics of runs 1 – 4. The number of stays per base (i.e., the number of detected changes in the amperage that do not correspond to new k-mers, above) and number of skips per base (i.e., the number of new k-mers in a basecalled sequence that do not correspond to the detected changes in amperage, below) for the four runs on the ISS and time-matched controls on the ground. The distributions were significantly different in all cases using the Kolmogorov-Smirnov test, but by so little where both runs were successful that there is likely no difference in signal inherent to data generated on the ISS vs. on the ground that would affect basecalling.

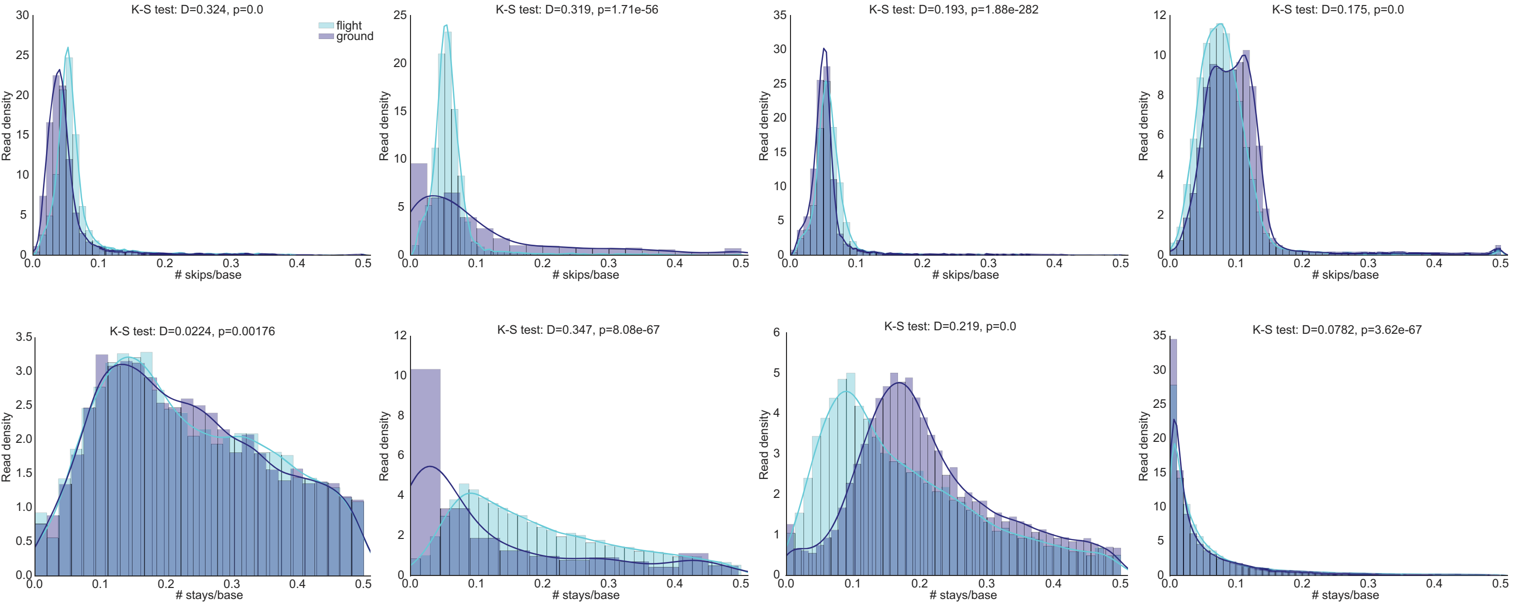
K-S test: D=0.324, p=0.0
K-S test: D=0.319, p=1.71e-56
K-S test: D=0.193, p=1.88e-282
K-S test: D=0.175, p=0.0
flight
ground
Read density
skips/base
K-S test: D=0.0224, p=0.00176
K-S test: D=0.347, p=8.08e-67
K-S test: D=0.219, p=0.0
K-S test: D=0.0782, p=3.62e-67
stays/base

Supplementary Figure 3. Alignment and base-calling statistics for ISS and Ground runs 1 – 4. The fraction identity of aligned segments for the reads generated on the ISS and on the ground divided by read type and species match. Legend: the 2D reads are shown for mouse, E. coli, and lambda, followed by the 1D reads of the template strand and the complement.

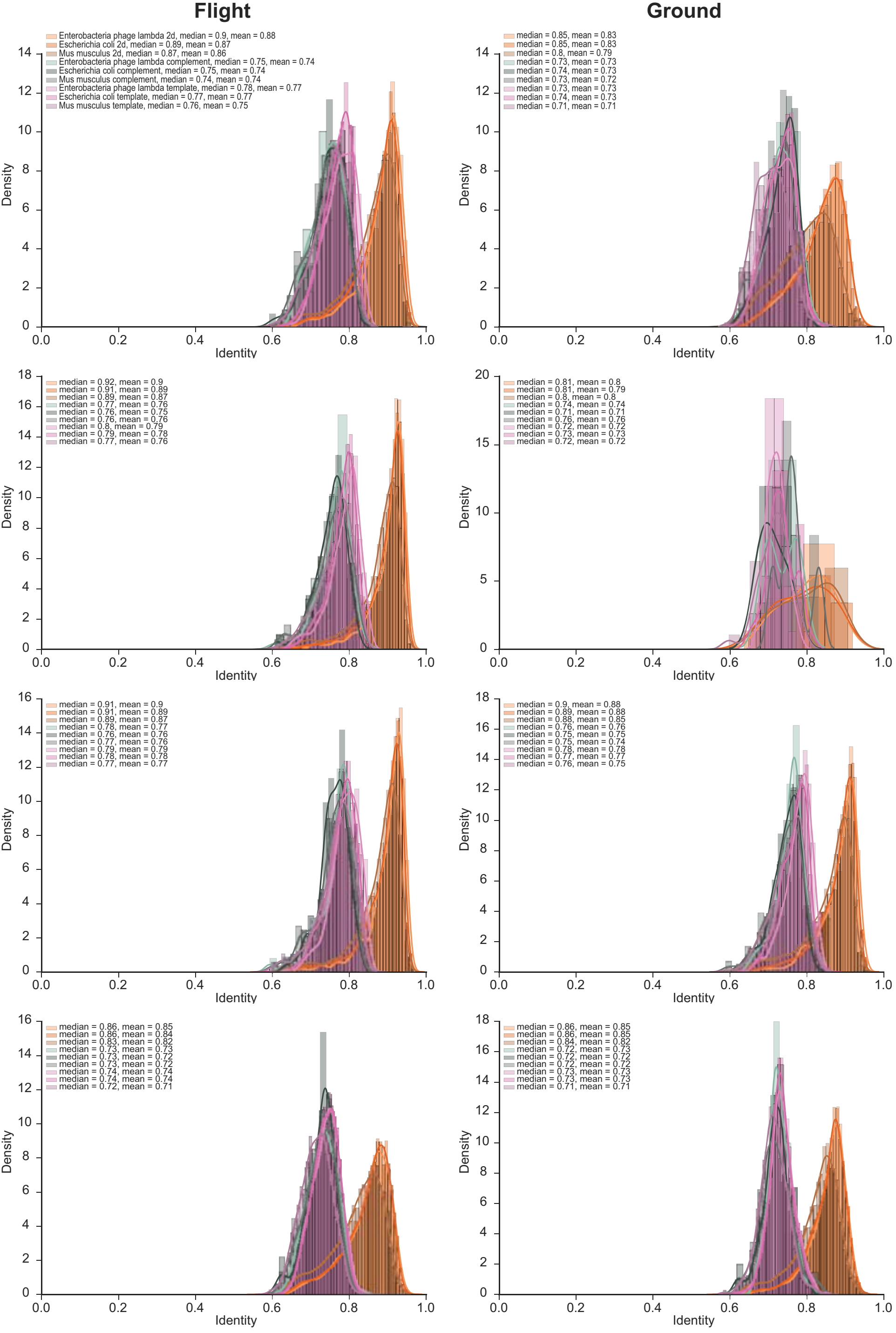
Flight
Ground
Enterobacteria phage lambda 2d, median = 0.9, mean = 0.88
Escherichia coli 2d, median = 0.89, mean = 0.87
Mus musculus 2d, median = 0.87, mean = 0.86
Enterobacteria phage lambda complement, median = 0.75, mean = 0.74
Escherichia coli complement, median = 0.75, mean = 0.74
Mus musculus complement, median = 0.74, mean = 0.74
Enterobacteria phage lambda template, median = 0.78, mean = 0.77
Escherichia coli template, median = 0.77, mean = 0.77
Mus musculus template, median = 0.76, mean = 0.75
median = 0.85, mean = 0.83
median = 0.85, mean = 0.83
median = 0.8, mean = 0.79
median = 0.73, mean = 0.73
median = 0.74, mean = 0.73
median = 0.73, mean = 0.72
median = 0.73, mean = 0.73
median = 0.74, mean = 0.73
median = 0.71, mean = 0.71
median = 0.92, mean = 0.9
median = 0.91, mean = 0.89
median = 0.89, mean = 0.87
median = 0.77, mean = 0.76
median = 0.76, mean = 0.75
median = 0.76, mean = 0.76
median = 0.8, mean = 0.79
median = 0.79, mean = 0.78
median = 0.77, mean = 0.76
median = 0.81, mean = 0.8
median = 0.81, mean = 0.79
median = 0.8, mean = 0.8
median = 0.74, mean = 0.74
median = 0.71, mean = 0.71
median = 0.76, mean = 0.76
median = 0.72, mean = 0.72
median = 0.73, mean = 0.73
median = 0.72, mean = 0.72
median = 0.91, mean = 0.9
median = 0.91, mean = 0.89
median = 0.89, mean = 0.87
median = 0.78, mean = 0.77
median = 0.76, mean = 0.76
median = 0.77, mean = 0.76
median = 0.79, mean = 0.79
median = 0.78, mean = 0.78
median = 0.77, mean = 0.77
median = 0.9, mean = 0.88
median = 0.89, mean = 0.88
median = 0.88, mean = 0.85
median = 0.76, mean = 0.76
median = 0.75, mean = 0.75
median = 0.75, mean = 0.74
median = 0.78, mean = 0.78
median = 0.77, mean = 0.77
median = 0.76, mean = 0.75
median = 0.86, mean = 0.85
median = 0.86, mean = 0.84
median = 0.83, mean = 0.82
median = 0.73, mean = 0.73
median = 0.73, mean = 0.72
median = 0.73, mean = 0.72
median = 0.74, mean = 0.74
median = 0.74, mean = 0.74
median = 0.72, mean = 0.71
median = 0.86, mean = 0.85
median = 0.86, mean = 0.85
median = 0.84, mean = 0.82
median = 0.72, mean = 0.73
median = 0.72, mean = 0.72
median = 0.72, mean = 0.72
median = 0.73, mean = 0.73
median = 0.73, mean = 0.73
median = 0.71, mean = 0.71
Density
Identity

Supplementary Figure 4. Coverage of the *E. coli* genome from MinION data in runs 1 – 4. Coverage for the *E. coli* genome across each run is plotted, sorted by date, showing the coverage (y-axis) across the genome length (x-axis). Alignments were done with the OneCodex platform.

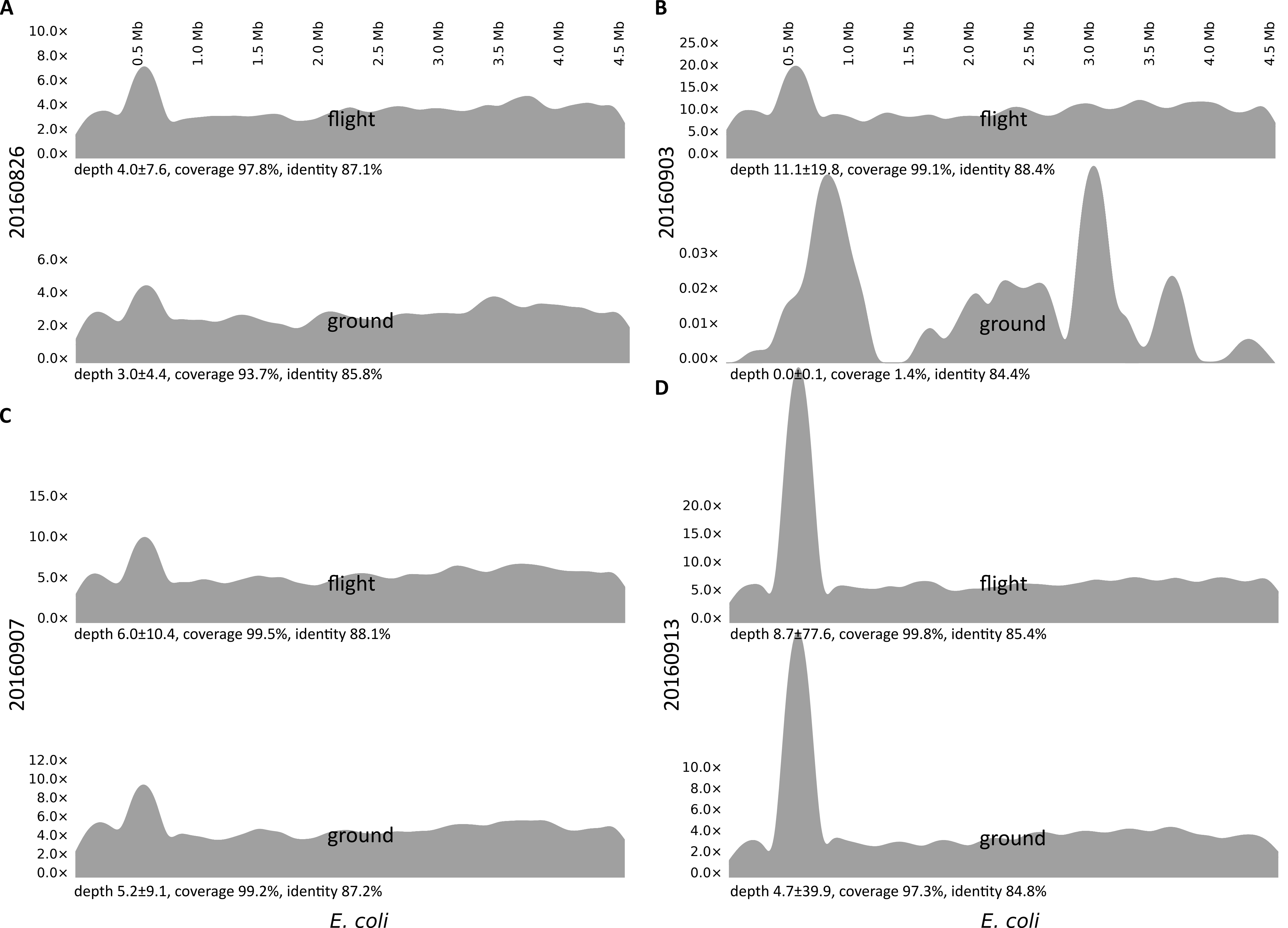
A
B
C
D
20160826
20160903
20160907
20160913
0.5 Mb
1.0 Mb
1.5 Mb
2.0 Mb
2.5 Mb
3.0 Mb
3.5 Mb
4.0 Mb
4.5 Mb
flight
ground
depth 4.0±7.6, coverage 97.8%, identity 87.1%
depth 3.0±4.4, coverage 93.7%, identity 85.8%
depth 11.1±19.8, coverage 99.1%, identity 88.4%
depth 0.0±0.1, coverage 1.4%, identity 84.4%
depth 6.0±10.4, coverage 99.5%, identity 88.1%
depth 5.2±9.1, coverage 99.2%, identity 87.2%
depth 8.7±77.6, coverage 99.8%, identity 85.4%
depth 4.7±39.9, coverage 97.3%, identity 84.8%
E. coli
E. coli

Supplementary Figure 5. Read lengths of ISS- and ground-based data. Read lengths divided by species after GraphMap alignment, for runs 1-4 (top to bottom), on the ISS (left) and on the ground (right). DNA were sheared using Covaris G-Tube standard protocols prior to library preparation, resulting in a distribution of fragment sizes.

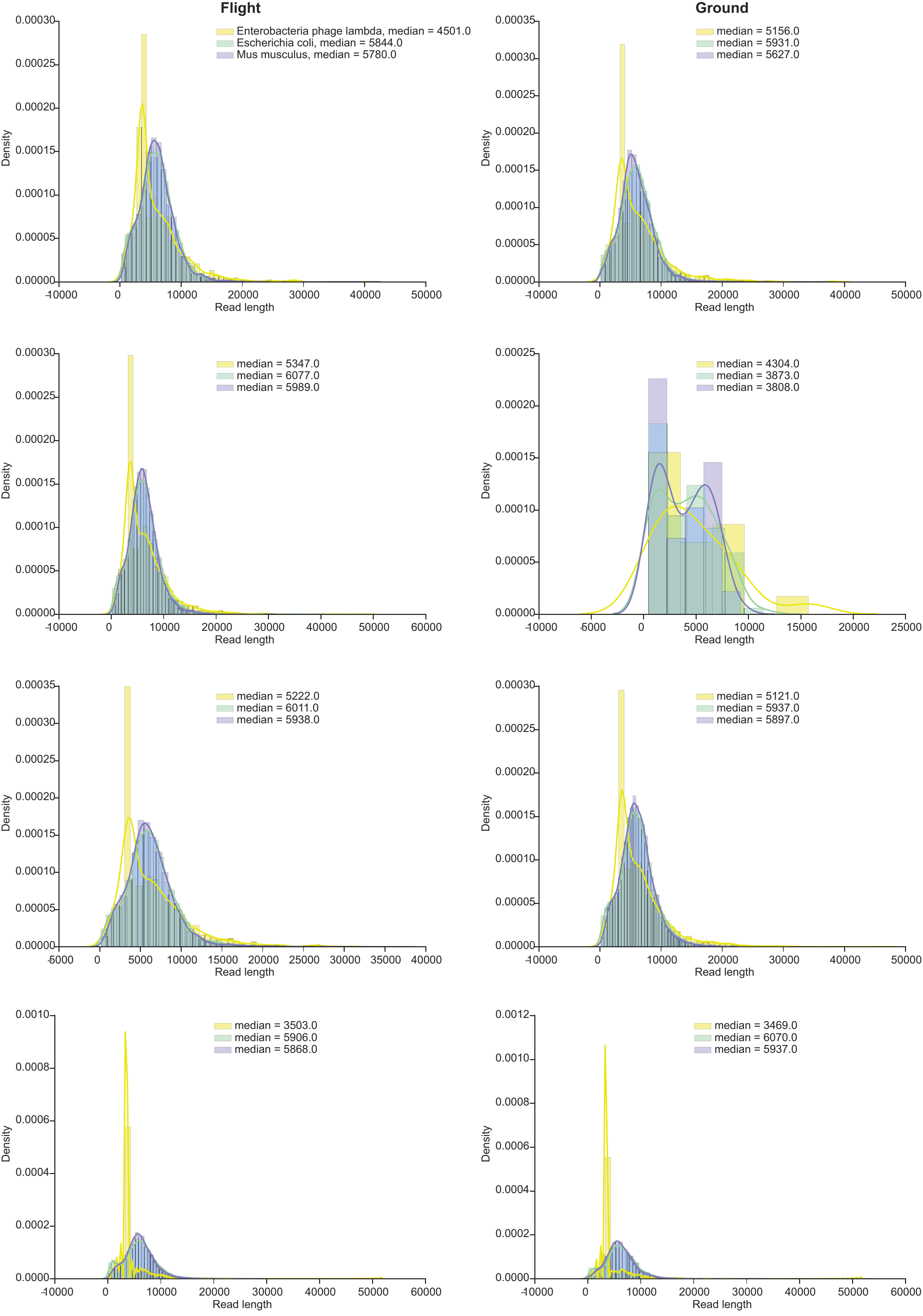
Flight
Ground
Enterobacteria phage lambda, median = 4501.0
Escherichia coli, median = 5844.0
Mus musculus, median = 5780.0
median = 5156.0
median = 5931.0
median = 5627.0
median = 5347.0
median = 6077.0
median = 5989.0
median = 4304.0
median = 3873.0
median = 3808.0
median = 5222.0
median = 6011.0
median = 5938.0
median = 5121.0
median = 5937.0
median = 5897.0
median = 3503.0
median = 5906.0
median = 5868.0
median = 3469.0
median = 6070.0
median = 5937.0
Density
Read length

Supplementary Figure 6. Coverage of the reference *E. coli* genome from the PacBio data. We observed an average of 162.7X coverage (y-axis) across the genome, which spanned the entire genome length (x-axis).

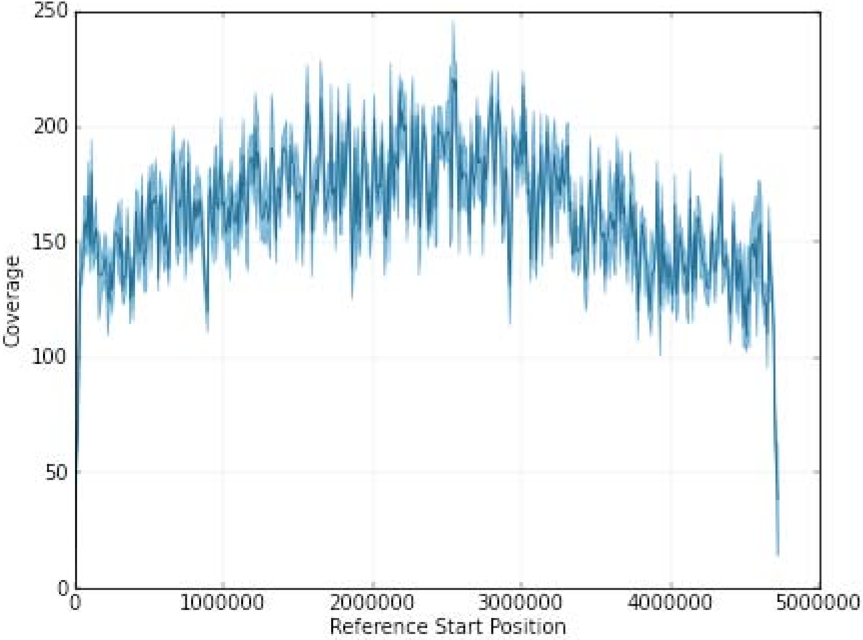

Coverage
Reference Start Position
0
50
100
150
200
250
0
1000000
2000000
3000000
4000000
5000000

Supplementary Figure 7: *De novo* genome assembly and cross-platform validation of the ISS nanopore run data. (A) *De novo* assembly of the *E. coli* genome from PacBio reads generates a single full-length contiguous sequence (contig) of length 4,734,145 base pairs (bp). (B) *De novo* assembly of the *E. coli* genome from ~9 million Illumina reads results in 245 mapped contigs (black segments) that assemble into a low-coverage, 80.1% complete genome (red bars) with 99.7% pairwise identity to the PacBio genome assembly. The orange bars denote regions of the genome with no coverage from an Illumina contig. **(C)** Direct assembly of the *E. coli* Illumina reads, identified by alignment to *E.* coli K-12, CP014348, to the PacBio genome assembly. As the *E. coli* CP014348 reference does not contain integrated lambda prophage, a narrow gap in coverage is observed corresponding to the lambda phage sequence inserted in the PacBio assembly ("lambda phage"). **(D)** Direct assembly of lambda Illumina reads to the lambda phage genome (LAMCG).

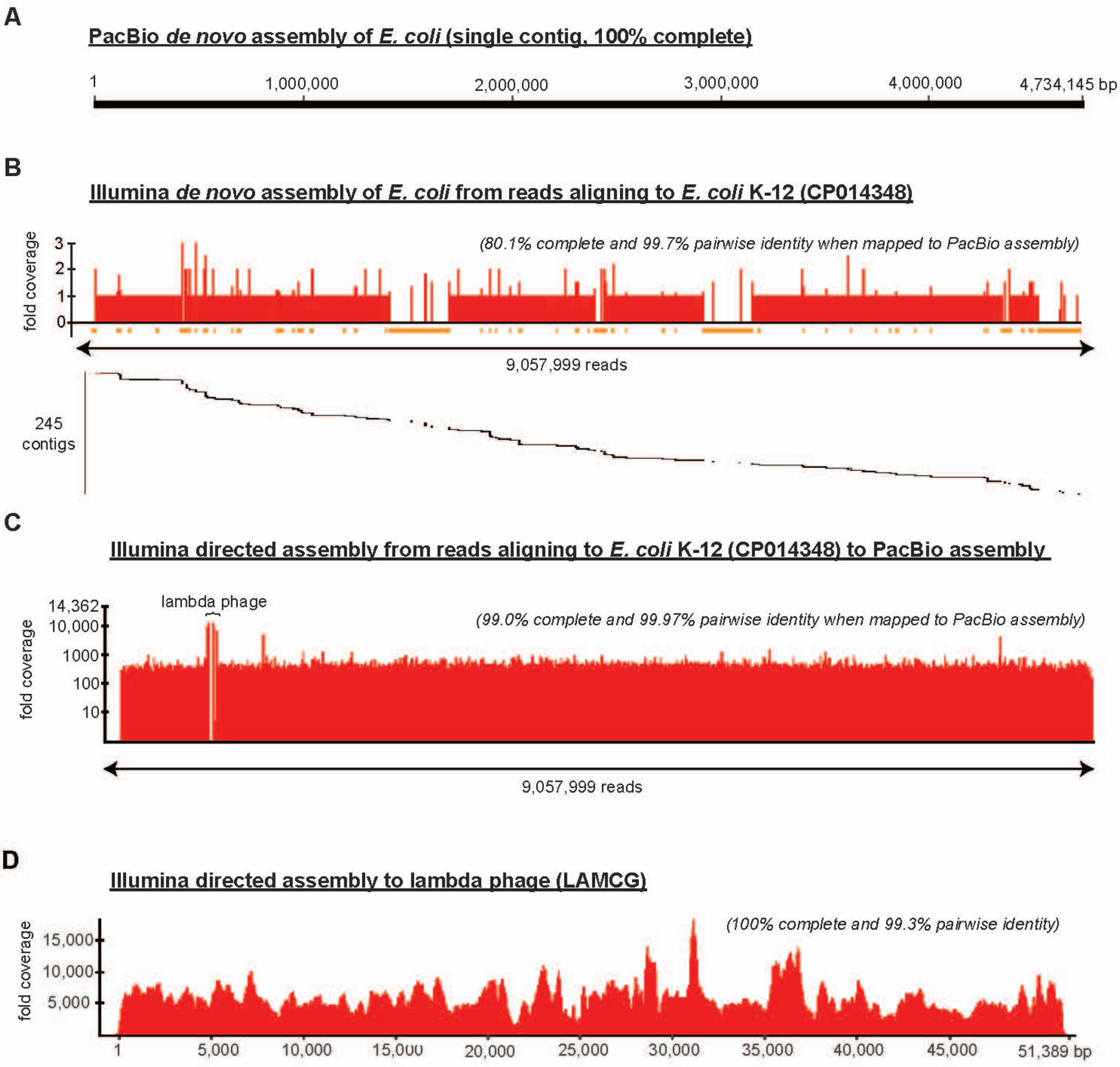
A
PacBio de novo assembly of E. coli (single contig, 100% complete)
1
1,000,000
2,000,000
3,000,000
4,000,000
4,734,145 bp
B
Illumina de novo assembly of E. coli from reads aligning to E. coli K-12 (CP014348)
fold coverage
3
2
1
0
(80.1% complete and 99.7% pairwise identity when mapped to PacBio assembly)
9,057,999 reads
245 contigs
C
Illumina directed assembly from reads aligning to E. coli K-12 (CP014348) to PacBio assembly
14,362
10,000
1000
100
10
fold coverage
lambda phage
(99.0% complete and 99.97% pairwise identity when mapped to PacBio assembly)
9,057,999 reads
D
Illumina directed assembly to lambda phage (LAMCG)
fold coverage
15,000
10,000
5,000
(100% complete and 99.3% pairwise identity)
1
5,000
10,000
15,000
20,000
25,000
30,000
35,000
40,000
45,000
51,389 bp

Supplementary Figure 8. *De novo* assembly of the *E. coli* genomes from in-flight ISS nanopore data, runs 1 – 8. Shown using Mauve software are alignments of *de novo* assembled contigs to the PacBio genome assembly used as a "gold standard" reference (gray background). **(A)** Contigs are *de novo* assembled using Miniasm from raw 2D reads (top panel), mouse-subtracted reads (middle panel), or *E. coli* reads (bottom panel). **(B)** Contigs are *de novo* assembled using Canu from raw 2D reads (top panel), mouse-subtracted reads (middle panel), or *E. coli* reads (bottom panel). Homologous segments are shown as colored blocks, with blocks that are shifted downward representing segments that are inverted relative to the PacBio genome assembly. Similarly colored lines connecting the blocks are used to indicate mapped positions in the reference genome.

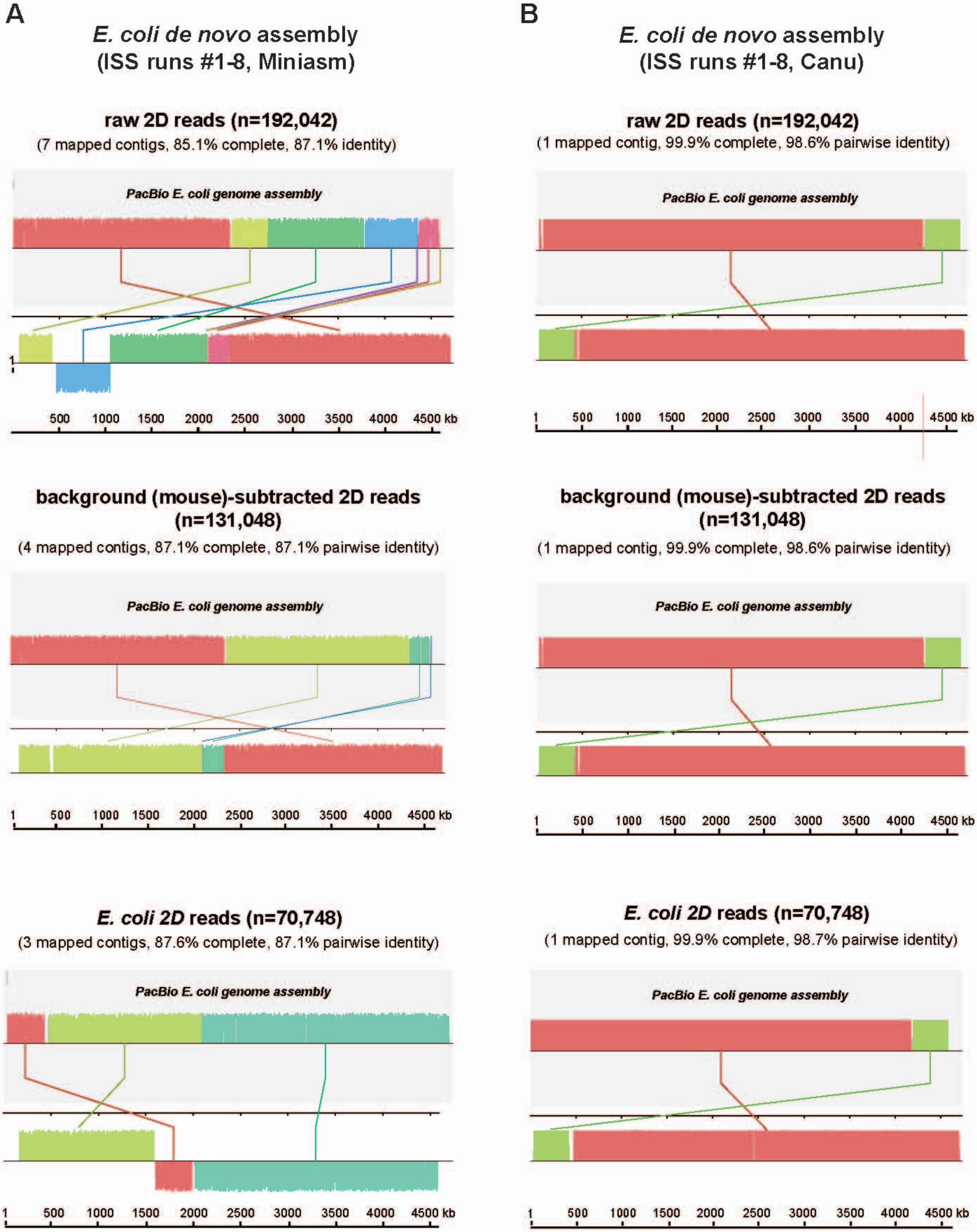
A
E. coli de novo assembly
(ISS runs #1-8, Miniasm)
raw 2D reads (n=192,042)
(7 mapped contigs, 85.1% complete, 87.1% identity)
PacBio E. coli genome assembly
500 1000 1500 2000 2500 3000 3500 4000 4500 kb
background (mouse)-subtracted 2D reads
(n=131,048)
(4 mapped contigs, 87.1% complete, 87.1% pairwise identity)
PacBio E. coli genome assembly
1 500 1000 1500 2000 2500 3000 3500 4000 4500 kb
E. coli 2D reads (n=70,748)
(3 mapped contigs, 87.6% complete, 87.1% pairwise identity)
PacBio E. coli genome assembly
1 500 1000 1500 2000 2500 3000 3500 4000 4500 kb
B
E. coli de novo assembly
(ISS runs #1-8, Canu)
raw 2D reads (n=192,042)
(1 mapped contig, 99.9% complete, 98.6% pairwise identity)
PacBio E. coli genome assembly
1 500 1000 1500 2000 2500 3000 3500 4000 4500 kb
background (mouse)-subtracted 2D reads
(n=131,048)
(1 mapped contig, 99.9% complete, 98.6% pairwise identity)
PacBio E. coli genome assembly
1 500 1000 1500 2000 2500 3000 3500 4000 4500 kb
E. coli 2D reads (n=70,748)
(1 mapped contig, 99.9% complete, 98.7% pairwise identity)
PacBio E. coli genome assembly
1 500 1000 1500 2000 2500 3000 3500 4000 4500 kb

Supplementary Figure 9. Cloud-based genome assembly. We used the Amazon Elastic Cloud Computing (EC2) platform to perform a *de novo* “miniasm” assembly of reads obtained from ISS runs 1 – 8, wherein we found that a 32GB RAM, 8-core processor instance could assemble the entire genome for E. coli in 15 seconds.

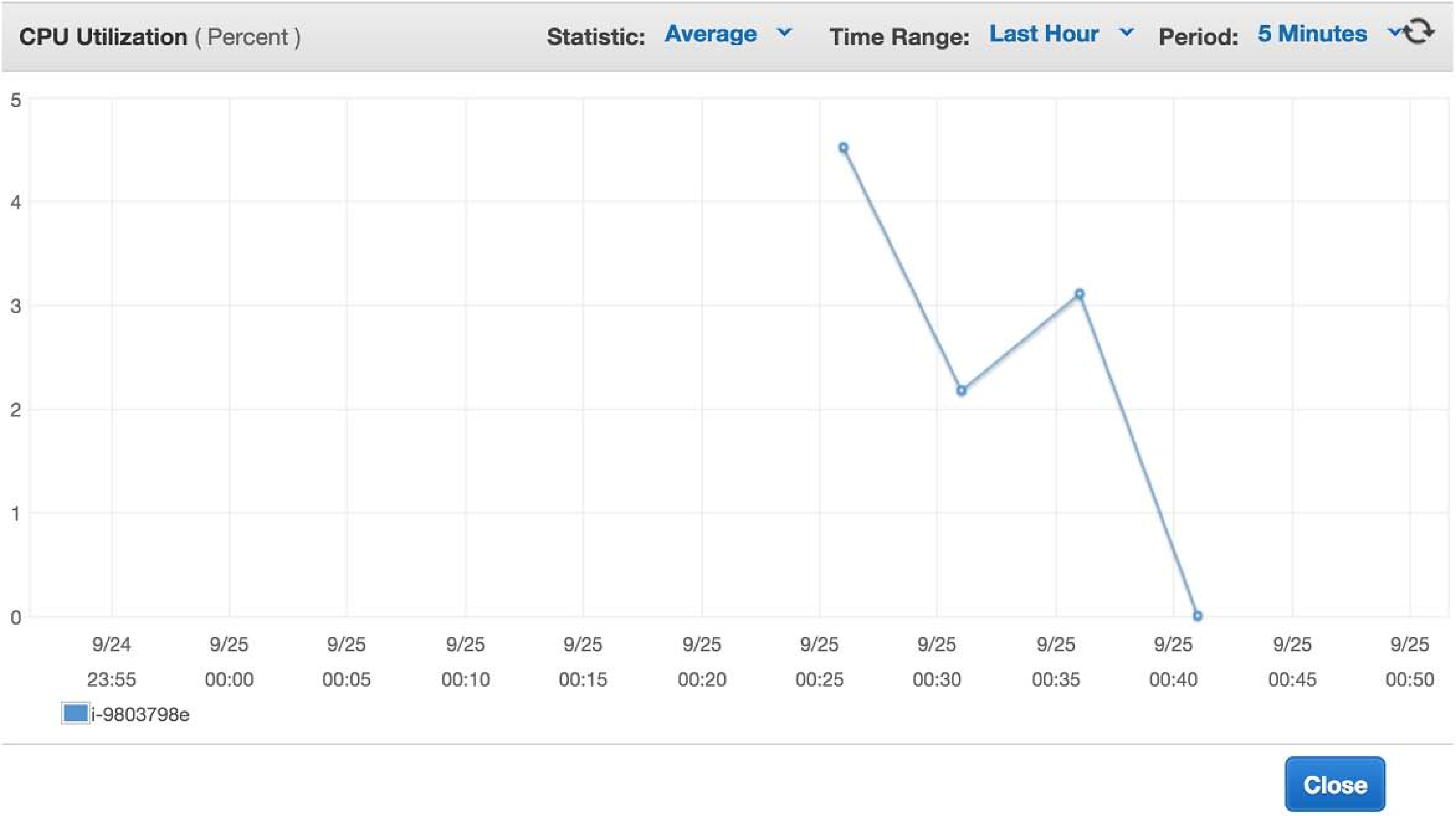

CPU Utilization (Percent)
Statistic: Average
Time Range: Last Hour
Period: 5 Minutes
5
4
3
2
1
0
9/24 23:55
9/25 00:00
9/25 00:05
9/25 00:10
9/25 00:15
9/25 00:20
9/25 00:25
9/25 00:30
9/25 00:35
9/25 00:40
9/25 00:45
9/25 00:50
i-9803798e
Close

Supplementary Figure 10. The Biomolecule Sequencer payload. (A) Surface Pro3 (B) MinION sequencer (C) USB 3.0 cable (D) R7.3 flow cell (E) empty sample syringe for air bubble removal (F) capped DNA containing sample syringe (G) outer transport tube for syringes and sample syringe tip.

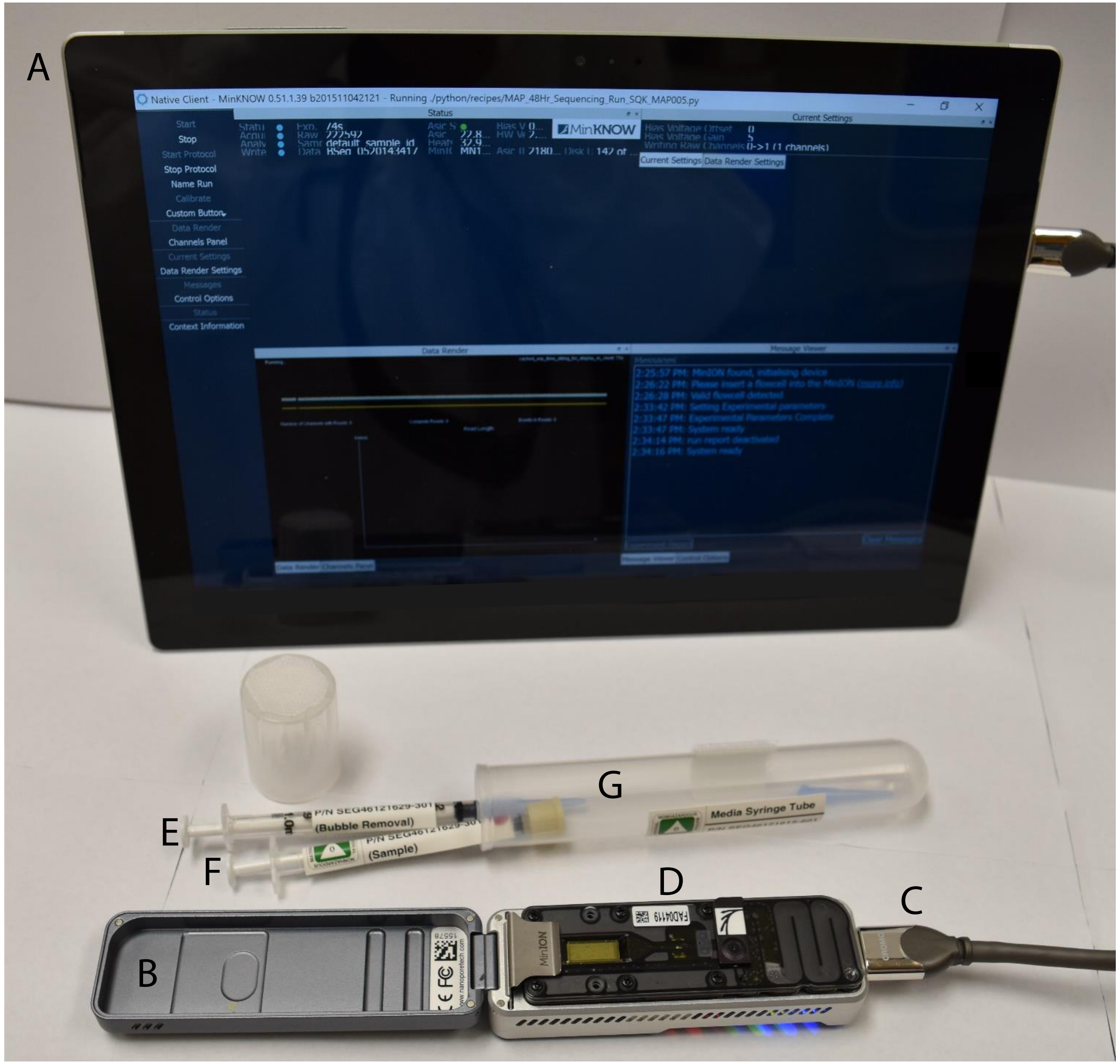
A
B
C
D
E
F
G
MinKNOW
Start
Stop
Start Protocol
Stop Protocol
Name Run
Calibrate
Custom Button
Data Render
Channels Panel
Current Settings
Data Render Settings
Messages
Control Options
Status
Context Information
(Bubble Removal)
(Sample)
Media Syringe Tube
MinION

Supplementary Figure 11. Computational workflow for the SURPIrt metagenomic analysis pipeline performed on data from ISS runs 1 – 8. Highlighted in red text are the reads identified ("+" branch) or remaining ("-" branch) after each step of the pipeline. Shown in the boxes are the megablast e-value cutoffs used for designating a positive hit ("E-value") and the number of matched hits (# matched hits") considered for taxonomic classification using the lowest common ancestor algorithm.

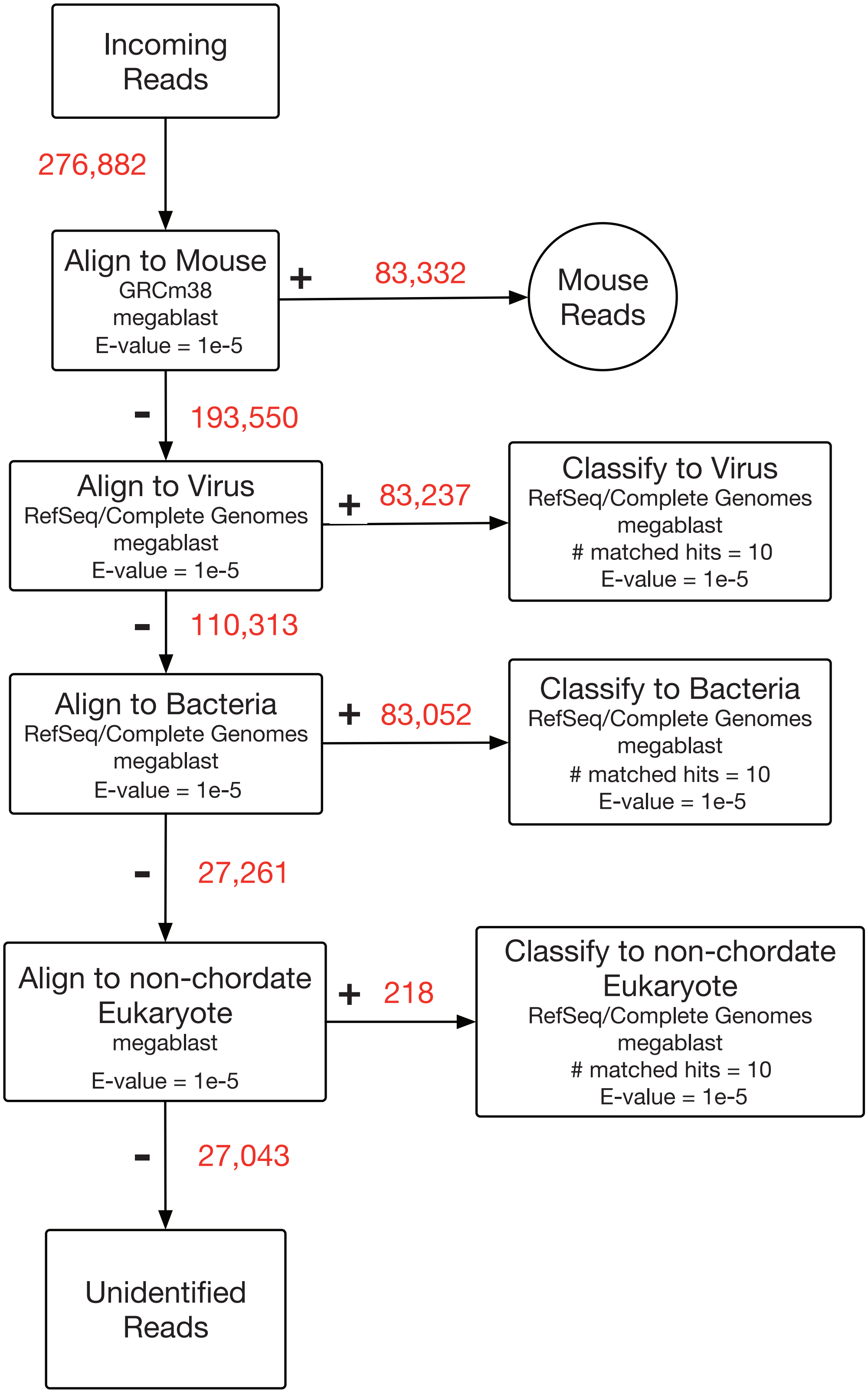
Incoming Reads
276,882
Align to Mouse
GRCm38
megablast
E-value = 1e-5
+
83,332
Mouse Reads
-
193,550
Align to Virus
RefSeq/Complete Genomes
megablast
E-value = 1e-5
+
83,237
Classify to Virus
RefSeq/Complete Genomes
megablast
matched hits = 10
E-value = 1e-5
-
110,313
Align to Bacteria
RefSeq/Complete Genomes
megablast
E-value = 1e-5
+
83,052
Classify to Bacteria
RefSeq/Complete Genomes
megablast
matched hits = 10
E-value = 1e-5
-
27,261
Align to non-chordate Eukaryote
megablast
E-value = 1e-5
+
218
Classify to non-chordate Eukaryote
RefSeq/Complete Genomes
megablast
matched hits = 10
E-value = 1e-5
-
27,043
Unidentified Reads

References

1 Li, H. Minimap: Experimental tool to find approximate mapping positions between long sequences. *https://github.com/lh3/minimap/* (2015).

2 Altschul, S. F., Gish, W., Miller, W., Myers, E. W. & Lipman, D. J. Basic local alignment search tool. *J Mol Biol* **215**, 403-410, doi:10.1016/S0022-2836(05)80360-2 (1990).
